# Increased WIC Cash Value Benefit is Associated with Greater Amount and Diversity of Redeemed Fruits and Vegetables among Participating Households

**DOI:** 10.1016/j.cdnut.2023.101986

**Published:** 2023-08-03

**Authors:** Christopher E. Anderson, Lauren E. Au, Catherine E. Yepez, Lorrene D. Ritchie, Marisa M. Tsai, Shannon E. Whaley

**Affiliations:** 1 Division of Research and Evaluation, Public Health Foundation Enterprises (PHFE) WIC, a program of Heluna Health, Irwindale, CA, United States; 2 Department of Nutrition, University of California Davis, Davis, CA, United States; 3 Nutrition Policy Institute, Division of Agriculture and Natural Resources, University of California, Oakland, CA, United States

**Keywords:** fruits, vegetables, WIC, nutrition assistance, fruit and vegetable access, fruit and vegetable diversity, nutrition policy

## Abstract

**Background:**

Special Supplemental Nutrition Program for Women, Infants, and Children (WIC) food packages for children ages 1 to 4 y include a cash value benefit (CVB) redeemable for fruits and vegetables (FVs) with participating vendors. The CVB value was increased beginning in June 2021.

**Objectives:**

This study evaluated associations of the augmented CVB with the amount and diversity of redeemed FVs.

**Methods:**

Price look-up codes (PLUs) in redemption data determined outcomes including *any redemption* (any, none), *amount redeemed* (United States dollars [USD]/mo), and *percent of total CVB redemption* (percent) in 54 FV commodity groups among a cohort of 1770 WIC-participating children in Southern California. Outcomes across all commodity groups for fresh fruits, fresh vegetables, and all FVs were evaluated including *dollar amount redeemed*, *percentage of redemption*, and *diversity of produce redeemed* (variety and balance among items redeemed). Comparisons were made between augmented CVB periods (35 USD/mo in June–September 2021, 24 USD/mo October 2021–June 2022) and the preaugment period (9 USD/mo in June 2020–May 2021). Associations were tested in multivariable generalized estimating equation Poisson (any redemption) and linear (amount, percent, diversity) regression models.

**Results:**

The augmented CVB was associated with higher *any redemption* prevalence and *amount redeemed* for 53 of 54 commodity groups at both 35 USD/mo and 24 USD/mo compared with 9 USD/mo. *Redemption diversity* increased for both fruits, vegetables, and all produce during both augment periods, and modestly greater increases in redeemed fruits relative to vegetables were observed at 35 USD/mo. The most commonly redeemed vegetables were tomatoes, onions, cucumbers, peppers, and avocados and the most commonly redeemed fruits were bananas, apples, grapes, limes, and melons.

**Conclusions:**

The augmented CVB was associated with greater redeemed FV amount and greater redeemed FV diversity. Data on FV intake diversity among WIC-participating children are needed to understand dietary impacts of the CVB increase.

## Introduction

Low diet quality is pervasive among young children in the United States [1], with notably low combined intakes of fruits and vegetables (FVs) [1,2] that are more pronounced among children living in low-income households [3]. Among children 1 to 4 y of age, average consumption of vegetables falls short of recommendations while consumption of fruit meets recommendations [1]. These shortfalls from healthy diets among children are comorbid with high and increasing obesity prevalence among young children [[4], [5], [6]] during an age period in which optimal nutrition is critical to both healthy growth and development of lifelong eating habits [7]. Both the amount and diversity of FVs consumed in childhood are important for health [8,9], but FVs are the foods most frequently rejected by young children [10], and greatly variable textures and flavors present challenges to getting children to eat produce that is not mild-flavored [11,12] and sweet [13]. Interventions to increase child FV intake have struggled to contribute to significant increases [14]. Increasing the amount of FVs available in households, which is associated with child FV intake [15], may help diversify and increase child FV intake.

The Special Supplemental Nutrition Program for Women, Infants, and Children (WIC) is a nutrition assistance program of the US federal government that provides support to pregnant and postpartum women living in low-income households and their infants and children under age 5 y with the aim of supporting healthy diets during these important life stages [16]. WIC provides 4 core services including food packages redeemable at approved vendors for select healthy foods and beverages, nutrition education, breastfeeding support, and health and social service referrals [17]. In 2021, WIC served 6.2 million people, including 3.4 million children aged 1 to 4 y [18]. WIC food packages were revised in 2009 to include a cash value benefit (CVB) for fresh FVs, in addition to canned and frozen FVs [19], and these food package changes were associated with significant improvements in the diets of participants [20,21], though diet quality among WIC-participating children has remained suboptimal [22,23].

In 2017, a National Academies of Science, Engineering, and Medicine committee issued recommendations for future revisions of the WIC food packages, recommending that the amount of the CVB for children ages 1 to 4 y be increased from 9 United States dollars (USD)/mo to 23 USD/mo, which would be enough to support approximately half of the intake of FVs recommended by the Dietary Guidelines for Americans [24,25]. In June of 2021, as part of the American Rescue Plan Act to mitigate the impacts of the COVID-19 pandemic, the WIC CVB was increased from 9 USD/mo to 35 USD/mo and was revised further via continuing resolution to 24 USD/mo from October 2021 to September 2022 [26,27]. The CVB was increased to 25 USD/mo for the period of October 1, 2022–September 30, 2023 and will expire without Congressional action. A proposed revision to the WIC food packages would make the enhanced CVB permanent [28], pending approval by the US Congress. Prior research identified positive perceptions of the augmented CVB among WIC-participating households in California [29], Delaware [30], North Carolina [31], and Massachusetts [32]; increases in food security and parental perception of child FV intake in California [33]; and increases in child FV intake nationally [34]. The objective of this study was to understand how both the amount and diversity of FVs redeemed at vendors using the WIC CVB changed following the introduction of the augmented benefit in June 2021 using redemption data and price look-up (PLU) codes among WIC-participating households in Los Angeles County, California. It was hypothesized that the augmented CVB would be associated with higher dollar amounts redeemed for FVs and greater diversity of FVs redeemed.

## Methods

### Setting and subjects

WIC participants served in 7 WIC sites by Public Health Foundation Enterprises WIC in Los Angeles County, California were invited to participate in a baseline survey in May 2021 that captured household sociodemographic information for this study. Only families with a WIC-participating child aged 1 to 4 y were eligible for inclusion in this study. Families with a complete survey in May 2021 and a follow-up survey in September 2021 and/or May 2022 were included in the final sample (*n*, children = 1770; *n*, families=1578), and redemption data were collected for these families from June 2020 to June 2022 (*n =* 22,440 observed months of redemption data at the family level). This observational study used data for all respondents who met the eligibility criteria. The California Health and Human Services Agency Committee for the Protection of Human Subjects provided Institutional Review Board approval for the study, and informed consent was provided by respondents with each survey.

### CVB periods

Redemption data were collected for participating families from June 2020 to June 2022 and grouped according to the amount of the CVB issued for child WIC participants ages 1 to 4 y. Household-level redemption of the WIC CVB was available monthly for participating families. Months between June 2020 and May 2021 were grouped together as Time 1 (T1, *n*, months of redemption data=12,032), when the CVB issued to each child was 9 USD/mo. Months between June 2021 and September 2021 were grouped together as Time 2 (T2, *n*, months of redemption = 4287), when the CVB issued to each child was 35 USD/mo. Months between October 2021 and June 2022 were grouped together as Time 3 (T3, *n*, months of redemption = 6121), when the CVB issued to each child was 24 USD/mo. These 3 time periods served as the independent variable of interest for every analysis in this study. All WIC benefit issuance and redemption data are available at the household level. Accordingly, CVB redemption evaluated in this study represents redemption of the aggregate CVB issued to the household (eg, if 2 children aged 1 to 4 y in a single household were issued a CVB in each month, they would receive 18 USD monthly in T1, 70 USD monthly in T2, and 48 USD monthly in T3).

### CVB redemption, commodity groups

Redemption of the CVB was assessed using WIC administrative data from electronic benefit transfer transactions with multiple outcomes for 54 specific commodity groups describing closely-related types of FVs based on the International Federation of Produce Standards categories [35], with PLUs aggregated to the commodity group level, and commodity groups were combined if there were fewer than 200 observed months of any redemption in that commodity group in the sample (Supplemental Table 1). Commodity groups were also aggregated into fresh fruits, fresh vegetables, and fresh (undetermined) FVs for retailer assigned PLU codes without a clear FV designation (labeled as “FOR USE WITH ALL COMMODITIES,” Supplemental Table 1). Finally, the amount of CVB redemption not accounted for by PLU-based redemption was categorized as “other fruit or vegetable” to capture redemption of non-PLU produce (eg, canned or frozen FV). The commodity group for berries was subsequently divided into individual types of berries (strawberries, blackberries, raspberries, blueberries) that had sufficient observed months (ie, ≥200 observed months) with any redemption. One outcome was *prevalence of any redemption* (any redemption > 0.00 USD, no redemption = 0.00 USD), determined for every family based upon whether any PLU codes for produce in specific commodity groups were redeemed each month. The second outcome was the *dollar amount redeemed* (USD), determined for every family based upon the total dollar amount redeemed for all PLU codes in the specified commodity groups each month. The third outcome was the *percent of PLU-based CVB redemption* (percent of USD redeemed for items with a PLU code) determined for every family as the percent of the total dollar amount redeemed across all PLU codes that were redeemed in the specified commodity groups each month.

### CVB redemption, summary measures

Aggregate measures were calculated for total FV redemption, incorporating both PLU and non-PLU redemption, for the *dollar amount redeemed* that were fresh fruits, fresh vegetables, fresh FVs (undetermined), and other (non-PLU) FVs (eg, canned or frozen fruits or vegetables), and the *percent of total CVB redemption* that was fresh fruits, fresh vegetables, fresh FVs (undetermined), and other (non-PLU) FVs. The proportion of total fresh produce that was redeemed as fruits was also calculated to determine the balance of fruits to vegetables redeemed.

Finally, *diversity of redemption* within fresh fruits, fresh vegetables, and all fresh produce were calculated. Diversity is a concept that encompasses disparity, variety, and balance within a population, and many metrics have been developed to capture this property of various populations [36]. Diversity scores were calculated to summarize disparity (ie, the different commodity groups that comprise FVs redeemed), variety (ie, the number of commodity groups redeemed), and balance (ie, proportions of redemption in separate commodity groups) of redemption across the commodity groups, with higher scores awarded for greater numbers of commodity groups redeemed and greater balance of redemption among the different redeemed commodity groups. The average number of commodity groups redeemed by a family each month for the study period was 6.5, so 0.1538 represents the sample average proportion redeemed in each commodity group assuming perfect balance (ie, 1 divided by 6.5 = 0.1538). A diversity score was calculated as follows: a score for each commodity group was determined by *step 1*) subtracting the proportion of CVB redemption in that commodity group from 0.1538 (measuring the distance from the average value of perfect balance in the population), *step 2*) taking the absolute value of this difference, *step 3*) adding 0.1 (to ensure that the maximum contribution of any single commodity group is truncated at 10 points), *step 4*) dividing 1 by the result from step 3, giving a commodity group-specific score. The overall diversity score was determined in *step 5*) by summing across all commodity groups. This process was conducted for all fresh produce (*n =* 54 commodity groups) and was repeated separately with commodity groups for fruit diversity (*n =* 24 commodity groups) and vegetable diversity (*n =* 30 commodity groups). This scoring process rewards both *1*) higher variety of redemption (ie, more commodity groups) and *2*) greater balance of redemption among redeemed commodity groups (ie, proportion of redemption being closer to 0.1538), while ensuring that no single commodity group contributes more than 10 points to the diversity score.

### Other variables

Respondent characteristics were assessed with WIC administrative data for the number of WIC-participating children in the household, child age (years), sex, and race/ethnicity and language preference (Asian, Black, Hispanic English-speaking, Hispanic Spanish-speaking, White, and Other). Survey responses were used to assess the number of children aged <18 y in the household, and household food insecurity at each survey using the 6-item USDA Household Food Security Survey Module and categorized as food insecure or not food insecure [37].

### Statistical analysis

Characteristics of children in the study were summarized with frequencies and percentages for categorical variables and means and standard deviations for continuous variables. Associations between the time period of CVB issuance (T2: 35 USD/mo and T3: 24 USD/mo compared with T1: 9 USD/mo) and each redemption outcome were tested in multivariable generalized estimating equation (GEE) regression models, accommodating clustering of multiple children and months of redemption data within households, with terms for independent variables including child race, sex, and age; household food insecurity and the number of household members under age 18; and calendar month for each month of redemption data (linear and quadratic) to control for seasonal variability in the availability of specific FVs. For associations between CVB period and any redemption in each commodity group, modified Poisson GEE regression models were used to determine prevalence rate ratios (PRR) and 95% CIs [38]. Associations between CVB period and dollar amount redeemed in each commodity group, for all fresh fruits, for all fresh vegetables, for all fresh FVs (undetermined), and for all other FVs (USD/mo), were calculated using linear GEE regression models to determine the change in redeemed dollar amount and 95% CI. Associations between CVB period and the percentage of PLU-based CVB redeemed in each commodity group, and percent of the CVB redeemed for all fresh fruits, for all fresh vegetables, for all fresh FVs (undetermined), for all other FVs, and for the percentage of all fresh FV redeemed as fruits, were calculated using linear GEE regression models to determine the change in percentage and 95% CI. Changes in diversity of redemption for fresh fruits, fresh vegetables, and all fresh produce and 95% CIs were calculated in linear GEE regression models. All analyses were conducted using SAS 9.4 (SAS Institute Inc.), and *P* values < 0.05 were considered statistically significant.

## Results

Over three-quarters of children included in the study were either English-speaking (45.1%) or Spanish-speaking (30.1%) Hispanic (Table 1), under half were female (47.0%), and children were an average of 2.76 y of age at the baseline survey in May 2021. At the baseline survey in May 2021, participating children lived in households with an average of 1.28 WIC-participating children aged 1 to 4 y, over one-third lived in households with 3 or more children under the age of 18 y (35.0%), and less than one half lived in a food secure household (46.0%). Children lived in households that redeemed an average of 91.2%, 89.4%, and 91.5% of the CVB issued during T1 (June 2020–May 2021), T2 (June 2021–September 2021), and T3 (October 2021–June 2022), respectively.

**TABLE 1:** Characteristics of WIC-participating Southern California children included in the study (*N =* 1770)

	Full sample *N =* 1770
Household has ≥3 children under age 18 y, *n* (%)	618 (35.0)
Household number of WIC-participating children aged 1–4 y, mean (SD)	1.3 (0.5)
Race/ethnicity, *n* (%)	
Asian	65 (3.7)
Black	222 (12.5)
Hispanic, English-speaking	798 (45.1)
Hispanic, Spanish-speaking	532 (30.1)
Other	114 (6.4)
White	39 (2.2)
Female, *n* (%)	832 (47.0)
Age (y), mean (SD)	2.8 (1.1)
Food secure household, n (%)	814 (46.0)

Abbreviation: WIC, Special Supplemental Nutrition Program for Women, Infants, and Children.

During T1 (June 2020–May 2021, 9 USD/mo CVB), vegetables with the highest monthly redemption prevalence were tomatoes (31.1%), peppers (17.9%), onions (17.7%), avocados (16.4%), and cucumbers (11.4%); these 5 categories remained the most prevalently redeemed at T2 (June 2021–September 2021, 35 USD/mo CVB: 59.1%, 45.2%, 47.7%, 40.1%, and 34.9%, respectively) and T3 (October 2021–June 2022, 24 USD/mo CVB: 54.0%, 37.6%, 41.5%, 29.6%, and 27.0%, respectively) (Table 2). Similarly, the vegetables that accounted for the highest monthly dollar amount redeemed at T1 were tomatoes (0.74 USD), avocados (0.47 USD), peppers (0.28 USD), onions (0.24 USD), and lettuce (0.22 USD); tomatoes, avocados, peppers, and onions remained the 4 highest redeemed, with squash replacing lettuce as the fifth highest value redeemed at T2 (2.13, 1.93, 0.98, 0.88, 0.84 USD, respectively) and potatoes replacing squash as the fifth highest value redeemed at T3 (1.93, 1.44, 0.88, 0.92, and 0.70, respectively) (Table 2). At T2 and T3, compared with T1, 29 of 30 evaluated vegetable categories exhibited significantly higher monthly prevalence of redemption (at T2, greatest increase for ginger root: PRR: 4.02, 95% CI: 3.14, 5.15; at T2, smallest increase for tomato: PRR: 1.96, 95% CI: 1.87, 2.05) and significantly higher dollar amount redeemed (at T2, greatest increase for avocados: 1.44 USD, 95% CI: 1.32, 1.56; at T2, smallest increase for kale: 0.03 USD, 95% CI: 0.01, 0.04). Changes were observed in the percent of PLU-based CVB redemption accounted for by many vegetables (Supplemental Table 2), with significant increases observed for avocados (T2 and T3), cabbage (T3 only), carrots (T2 only), garlic (T3 only), kale (T2 and T3), leafy greens (T3 only), onions (T2 and T3), other root vegetables (T3 only), peppers (T3 only), potatoes (T2 and T3), and squash (T2 and T3) compared with T1; significant decreases were observed for asparagus (T3 only), lettuce (T2 only), and tomatoes (T2 only) compared with T1.

**TABLE 2:** Redemption of vegetables by WIC-participating households in Southern California before and during augmentation of the CVB for fruits and vegetables, June 2020–June 2022

	Any redemption prevalence					Dollars redeemed				
	Prevalence (%)^1^			Association with CVB period^2^		Dollars, mean (SD)^3^			Association with CVB period^4^	
	T1	T2	T3	T2 vs. T1	T3 vs. T1	T1	T2	T3	T2 vs. T1	T3 vs. T1
Asparagus	2.2	4.2	3.1	2.43 (1.97, 3.00)	1.40 (1.15, 1.70)	0.06 (0.49)	0.18 (1.04)	0.11 (0.70)	0.14 (0.10, 0.17)	0.05 (0.02, 0.07)
Avocados	16.4	40.1	29.6	2.50 (2.34, 2.67)	1.84 (1.71, 1.97)	0.47 (1.30)	1.93 (3.30)	1.44 (2.89)	1.44 (1.32, 1.56)	0.95 (0.84, 1.06)
Beans	3.7	9.6	8.3	2.90 (2.46, 3.41)	2.22 (1.91, 2.59)	0.08 (0.54)	0.25 (1.11)	0.20 (0.92)	0.18 (0.13, 0.22)	0.11 (0.08, 0.14)
Beets	0.9	2.8	1.9	3.64 (2.66, 4.98)	2.32 (1.71, 3.14)	0.01 (0.19)	0.06 (0.44)	0.04 (0.32)	0.04 (0.03, 0.06)	0.02 (0.01, 0.03)
Broccoli	6.8	18.8	13.2	2.79 (2.50, 3.12)	2.09 (1.86, 2.34)	0.12 (0.55)	0.42 (1.18)	0.28 (0.92)	0.30 (0.25, 0.34)	0.17 (0.14, 0.21)
Brussels sprouts	0.4	1.0	1.0	4.00 (2.45, 6.51)	2.49 (1.55, 4.00)	0.01 (0.14)	0.04 (0.44)	0.03 (0.33)	0.03 (0.01, 0.05)	0.02 (0.01, 0.03)
Cabbage	4.6	13.5	12.7	3.05 (2.67, 3.49)	2.90 (2.56, 3.28)	0.07 (0.40)	0.27 (0.86)	0.23 (0.75)	0.19 (0.16, 0.22)	0.16 (0.14, 0.19)
Carrots	5.6	17.6	14.4	3.43 (3.06, 3.84)	2.60 (2.29, 2.95)	0.07 (0.36)	0.24 (0.69)	0.20 (0.63)	0.18 (0.16, 0.21)	0.13 (0.10, 0.15)
Cauliflower	2.0	5.9	4.6	2.98 (2.41, 3.69)	2.43 (1.98, 2.98)	0.06 (0.47)	0.17 (0.81)	0.15 (0.75)	0.12 (0.09, 0.15)	0.09 (0.07, 0.11)
Celery	4.1	11.7	9.1	3.16 (2.72, 3.67)	2.37 (2.06, 2.72)	0.07 (0.45)	0.21 (0.82)	0.17 (0.73)	0.15 (0.12, 0.18)	0.10 (0.08, 0.13)
Chard	0.6	1.0	0.9	1.52 (0.96, 2.40)	1.86 (1.18, 2.95)	0.01 (0.20)	0.03 (0.35)	0.03 (0.48)	0.01 (-0.00, 0.03)	0.02 (0.00, 0.03)
Corn	5.9	17.5	12.1	3.07 (2.74, 3.44)	2.10 (1.87, 2.36)	0.14 (0.67)	0.48 (1.38)	0.38 (1.32)	0.35 (0.30, 0.40)	0.23 (0.19, 0.28)
Cucumbers	11.9	34.9	27.0	2.85 (2.64, 3.07)	2.34 (2.17, 2.53)	0.18 (0.63)	0.69 (1.29)	0.51 (1.12)	0.48 (0.43, 0.53)	0.34 (0.29, 0.38)
Garlic	3.2	10.3	9.0	3.36 (2.90, 3.89)	2.96 (2.55, 3.43)	0.05 (0.34)	0.18 (0.64)	0.19 (0.74)	0.13 (0.11, 0.15)	0.14 (0.11, 0.16)
Ginger root	1.3	4.4	3.3	4.02 (3.14, 5.15)	2.47 (1.96, 3.12)	0.02 (0.28)	0.06 (0.45)	0.05 (0.45)	0.04 (0.03, 0.06)	0.02 (0.00, 0.04)
Kale	0.5	1.7	1.2	3.54 (2.20, 5.68)	2.75 (1.69, 4.46)	0.01 (0.12)	0.03 (0.45)	0.02 (0.22)	0.03 (0.01, 0.04)	0.02 (0.01, 0.02)
Leafy green	1.2	3.5	3.5	3.23 (2.44, 4.28)	3.15 (2.46, 4.03)	0.03 (0.34)	0.10 (0.64)	0.10 (0.68)	0.07 (0.05, 0.10)	0.07 (0.05, 0.10)
Lettuce	11.1	28.4	21.7	2.50 (2.30, 2.72)	1.99 (1.83, 2.18)	0.22 (0.79)	0.67 (1.58)	0.53 (1.34)	0.44 (0.38, 0.50)	0.32 (0.27, 0.37)
Mushrooms	3.2	7.6	5.7	2.66 (2.27, 3.12)	1.82 (1.54, 2.16)	0.08 (0.51)	0.23 (0.96)	0.17 (0.82)	0.16 (0.13, 0.19)	0.10 (0.07, 0.13)
Non-leafy green	0.3	1.3	1.0	3.73 (2.39, 5.81)	3.08 (1.91, 4.97)	0.01 (0.23)	0.05 (0.50)	0.03 (0.39)	0.04 (0.02, 0.06)	0.02 (0.01, 0.04)
Onions	17.7	47.7	41.5	2.87 (2.70, 3.06)	2.40 (2.25, 2.56)	0.24 (0.68)	0.88 (1.36)	0.92 (1.59)	0.68 (0.63, 0.73)	0.69 (0.63, 0.76)
Other root vegetable	2.2	5.3	6.0	2.94 (2.41, 3.58)	2.79 (2.32, 3.35)	0.04 (0.36)	0.11 (0.59)	0.16 (0.80)	0.09 (0.06, 0.11)	0.12 (0.09, 0.15)
Other vegetable	2.0	6.1	5.4	3.34 (2.66, 4.18)	2.68 (2.15, 3.34)	0.07 (0.58)	0.22 (1.29)	0.19 (1.12)	0.16 (0.11, 0.21)	0.11 (0.08, 0.15)
Peppers	17.9	45.2	37.6	2.64 (2.48, 2.80)	2.13 (1.99, 2.28)	0.28 (0.85)	0.98 (1.70)	0.88 (1.78)	0.71 (0.65, 0.77)	0.60 (0.53, 0.66)
Potato	8.3	25.6	21.8	3.30 (3.01, 3.61)	2.63 (2.40, 2.89)	0.19 (0.72)	0.69 (1.48)	0.70 (1.59)	0.52 (0.47, 0.58)	0.51 (0.45, 0.57)
Radish	2.3	7.2	6.2	3.15 (2.63, 3.78)	2.60 (2.16, 3.14)	0.02 (0.16)	0.07 (0.32)	0.07 (0.35)	0.05 (0.04, 0.06)	0.05 (0.04, 0.06)
Spinach	2.2	5.8	4.3	2.81 (2.25, 3.52)	1.87 (1.51, 2.32)	0.04 (0.34)	0.13 (0.68)	0.09 (0.57)	0.10 (0.07, 0.13)	0.05 (0.03, 0.07)
Squash	11.4	31.5	25.8	3.06 (2.82, 3.32)	2.29 (2.11, 2.48)	0.19 (0.66)	0.84 (1.86)	0.60 (1.37)	0.67 (0.61, 0.74)	0.41 (0.36, 0.46)
Sweet potato	2.5	7.4	6.3	3.28 (2.73, 3.92)	2.32 (1.94, 2.79)	0.05 (0.41)	0.20 (0.90)	0.19 (0.96)	0.15 (0.12, 0.18)	0.13 (0.09, 0.17)
Tomato	31.1	59.1	54.0	1.96 (1.87, 2.05)	1.76 (1.68, 1.84)	0.74 (1.44)	2.13 (2.77)	1.93 (2.93)	1.42 (1.31, 1.52)	1.18 (1.06, 1.30)

Abbreviations: CVB, cash value benefit; SD, standard deviation; WIC, Special Supplemental Nutrition Program for Women, Infants and Children.

1 Any redemption is expressed as the percentage of all food benefit obligation months in which there was any redemption in the specified category of vegetables during the 3 CVB amounts issued (T1: 9 USD/mo; T2: 35 USD/mo; T3: 24 USD/mo) during the study period. Number of observed months of redemption data in each study period are the denominator for each calculation (T1: 12,032 mo; T2: 4287 mo; T3: 6121 mo).

2 Prevalence rate ratio (95% confidence interval) for any redemption of specified category of vegetables was determined for T2 (35 USD/mo) and T3 (24 USD/mo) compared with T1 (9 USD/mo) in generalized estimating equations modified Poisson regression models adjusted for child race/ethnicity, sex, and age; household food insecurity and the number of household members under age 18; and calendar month (linear and quadratic). Models also accommodated clustering of monthly observations within participating children and families.

3 Dollar amount is expressed as the mean (SD) amount of CVB redeemed per family (USD) in the specified category of vegetables for months during the 3 CVB amounts issued (T1: 9 USD/mo; T2: 35 USD/mo; T3: 24 USD/mo) during the study period. Every observed month of redemption data was used in the calculation of means and SDs during each study period (T1: 12,032 mo; T2: 4287 mo; T3: 6121 mo).

4 Estimate (95% confidence interval) for USD redeemed of specified category of vegetables was determined for T2 (35 USD/mo) and T3 (24 USD/mo) compared with T1 (9 USD/mo) in generalized estimating equations linear regression models adjusted for child race/ethnicity, sex, and age; household food insecurity and the number of household members under age 18; and calendar month (linear and quadratic). Models also accommodated clustering of monthly observations within participating children and families.

At baseline, fruits with the highest monthly redemption prevalence were bananas (37.4%), apples (15.5%), limes (13.0%), grapes (12.2%), and melon (10.6%); these 5 categories remained the most prevalently redeemed at T2 (65.2%, 36.4%, 32.6%, 31.3%, and 34.3%, respectively) and T3 (55.0%, 37.0%, 22.7%, 22.1%, and 21.0%, respectively) (Table 3). Similarly, the fruits that accounted for the highest monthly dollar amount redeemed at T1 were bananas (0.80 USD), grapes (0.46 USD), apples (0.39 USD), melon (0.41 USD), and strawberries (0.29 USD); these 5 categories continued to account for the highest dollar amount redeemed at T2 (2.08, 1.60, 1.39, 1.89, and 1.01 USD, respectively) and T3 (1.61, 1.30, 1.49, 1.27, and 0.91 USD, respectively) (Table 3). Significantly higher monthly redemption prevalence (at T2, greatest increase for blackberries: PRR: 5.08, 95% CI: 3.09, 8.35; at T2, smallest increase for banana: PRR: 1.77, 95% CI: 1.70, 1.84) and dollar amount redeemed (at T2, greatest increase for mango: 1.35 USD, 95% CI: 1.21, 1.50; at T2, smallest increase for blackberries: 0.03 USD, 95% CI, 0.01, 0.04) were observed for all 24 evaluated fruit categories at T2 and T3 compared with T1. Changes were observed in the percent of PLU-based CVB redemption accounted for by many fruits (Supplemental Table 3), with significant increases observed for apples (T3 only), lemons (T2 and T3), mango (T2 and T3), nectarines (T2 only), other fruits (T2 only), peaches (T2 only), raspberries (T2 only), and strawberries (T3 only), and significant decreases observed for bananas (T2 and T3) and limes (T2 only) compared with T1.

**TABLE 3:** Redemption of fruits by WIC-participating households in Southern California before and during augmentation of the CVB for fruits and vegetables, June 2020–June 2022

	Any redemption prevalence					Dollars redeemed				
	Prevalence (%)^1^			Association with CVB period^2^		Dollars, mean (SD)^3^			Association with CVB period^4^	
	T1	T2	T3	T2 vs. T1	T3 vs. T1	T1	T2	T3	T2 vs. T1	T3 vs. T1
Apples	15.5	36.4	37.0	2.48 (2.32, 2.66)	2.41 (2.24, 2.59)	0.39 (1.11)	1.39 (2.48)	1.49 (2.69)	1.04 (0.94, 1.13)	1.10 (0.98, 1.21)
Bananas	37.4	65.2	55.0	1.77 (1.70, 1.84)	1.49 (1.42, 1.55)	0.80 (1.35)	2.08 (2.45)	1.61 (2.25)	1.30 (1.20, 1.39)	0.84 (0.74, 0.94)
Blackberries	0.3	1.2	1.0	5.08 (3.09, 8.35)	2.70 (1.62, 4.49)	0.01 (0.17)	0.03 (0.36)	0.04 (0.46)	0.03 (0.01, 0.04)	0.03 (0.01, 0.04)
Blueberries	1.5	4.2	2.8	2.24 (1.78, 2.82)	1.75 (1.37, 2.24)	0.05 (0.50)	0.17 (1.01)	0.10 (0.72)	0.10 (0.06, 0.13)	0.05 (0.02, 0.07)
Cherries	1.7	9.7	3.3	4.28 (3.52, 5.20)	1.99 (1.62, 2.44)	0.08 (0.71)	0.52 (1.93)	0.22 (1.37)	0.37 (0.30, 0.44)	0.15 (0.11, 0.19)
Dragon fruit	0.4	1.4	0.9	3.82 (2.38, 6.14)	2.96 (1.91, 4.59)	0.02 (0.39)	0.11 (1.11)	0.07 (0.84)	0.09 (0.05, 0.13)	0.05 (0.03, 0.08)
Grapes	12.2	31.3	22.1	2.57 (2.39, 2.77)	1.79 (1.65, 1.95)	0.46 (1.44)	1.60 (3.06)	1.30 (2.99)	1.20 (1.09, 1.32)	0.80 (0.69, 0.91)
Kiwifruit	2.6	7.8	5.2	2.98 (2.51, 3.54)	1.97 (1.63, 2.38)	0.05 (0.40)	0.28 (1.38)	0.15 (0.75)	0.21 (0.16, 0.25)	0.09 (0.07, 0.12)
Lemons	3.0	9.4	8.1	3.28 (2.79, 3.86)	2.86 (2.41, 3.40)	0.06 (0.44)	0.25 (0.93)	0.20 (0.86)	0.19 (0.16, 0.23)	0.14 (0.11, 0.18)
Limes	13.0	32.6	22.7	2.42 (2.25, 2.60)	1.79 (1.66, 1.93)	0.27 (0.91)	0.81 (1.67)	0.76 (2.08)	0.57 (0.51, 0.63)	0.46 (0.38, 0.53)
Mango	9.0	26.1	19.5	2.57 (2.34, 2.81)	2.33 (2.13, 2.54)	0.22 (0.85)	0.98 (2.17)	0.76 (2.05)	0.72 (0.64, 0.81)	0.55 (0.48, 0.63)
Melon	10.6	34.3	21.0	2.65 (2.45, 2.87)	2.10 (1.92, 2.29)	0.41 (1.43)	1.89 (3.72)	1.27 (3.25)	1.35 (1.21, 1.50)	0.89 (0.77, 1.01)
Nectarine	3.2	18.7	6.5	5.04 (4.37, 5.82)	1.99 (1.71, 2.32)	0.09 (0.59)	0.59 (1.69)	0.25 (1.15)	0.48 (0.41, 0.55)	0.16 (0.12, 0.19)
Oranges	8.0	18.4	15.3	2.27 (2.06, 2.52)	1.97 (1.77, 2.18)	0.21 (0.86)	0.58 (1.53)	0.50 (1.51)	0.35 (0.30, 0.41)	0.30 (0.25, 0.36)
Other citrus	1.1	3.0	1.9	3.00 (2.25, 3.99)	1.58 (1.17, 2.13)	0.03 (0.37)	0.10 (0.68)	0.06 (0.50)	0.07 (0.05, 0.10)	0.03 (0.01, 0.04)
Other fruit	1.8	7.5	4.2	3.68 (3.02, 4.49)	2.38 (1.95, 2.90)	0.05 (0.45)	0.29 (1.51)	0.16 (1.05)	0.23 (0.17, 0.28)	0.11 (0.08, 0.15)
Papaya	3.6	10.0	7.7	2.69 (2.30, 3.14)	2.20 (1.88, 2.57)	0.11 (0.66)	0.37 (1.37)	0.28 (1.14)	0.24 (0.19, 0.29)	0.17 (0.13, 0.21)
Peaches	2.9	16.3	5.6	4.01 (3.44, 4.67)	2.08 (1.76, 2.47)	0.08 (0.54)	0.51 (1.48)	0.19 (0.95)	0.39 (0.34, 0.45)	0.12 (0.09, 0.15)
Pears	4.2	11.1	9.2	2.77 (2.41, 3.17)	2.11 (1.82, 2.45)	0.09 (0.53)	0.29 (1.00)	0.26 (1.01)	0.21 (0.17, 0.25)	0.16 (0.13, 0.20)
Pineapple	2.8	8.5	5.6	3.15 (2.65, 3.75)	1.96 (1.65, 2.33)	0.09 (0.59)	0.34 (1.30)	0.20 (0.96)	0.25 (0.20, 0.30)	0.11 (0.08, 0.14)
Plums	1.8	7.0	2.6	2.71 (2.21, 3.32)	1.64 (1.30, 2.08)	0.04 (0.32)	0.16 (0.71)	0.08 (0.62)	0.11 (0.08, 0.13)	0.05 (0.03, 0.07)
Raspberries	1.0	3.2	2.6	4.29 (3.25, 5.66)	2.12 (1.64, 2.74)	0.03 (0.36)	0.13 (0.82)	0.11 (0.87)	0.12 (0.09, 0.15)	0.07 (0.05, 0.10)
Strawberries	8.1	22.6	19.2	2.73 (2.47, 3.01)	2.49 (2.26, 2.75)	0.29 (1.12)	1.01 (2.36)	0.91 (2.30)	0.76 (0.67, 0.85)	0.64 (0.55, 0.72)
Tangerine/mandarin	4.1	8.4	8.0	2.64 (2.25, 3.10)	1.85 (1.60, 2.14)	0.12 (0.69)	0.33 (1.32)	0.35 (1.38)	0.24 (0.19, 0.29)	0.22 (0.17, 0.27)

Abbreviations: CVB, cash value benefit; USD, United States dollars; WIC, Special Supplemental Nutrition Program for Women, Infants and Children.

1 Any redemption is expressed as the percentage of all months in which there was any redemption in the specified category of fruits during the 3 CVB amounts issued (T1: 9 USD/mo; T2: 35 USD/mo; T3: 24 USD/mo) during the study period. Number of observed months of redemption data in each study period are the denominator for each calculation (T1: 12,032 mo; T2: 4287 mo; T3: 6121 mo).

2 Prevalence rate ratio (95% confidence interval) for any redemption of specified category of fruits was determined for T2 (35 USD/mo) and T3 (24 USD/mo) compared with T1 (9 USD/mo) in generalized estimating equations modified Poisson regression models adjusted for child race/ethnicity, sex, and age; household food insecurity and the number of household members under age 18; and calendar month (linear and quadratic). Models also accommodated clustering of monthly observations within participating children and families.

3 Dollar amount is expressed as the mean (SD) amount of CVB redeemed per family (USD) in the specified category of fruits for months during the 3 CVB amounts issued (T1: 9 USD/mo; T2: 35 USD/mo; T3: 24 USD/mo) during the study period. Every observed month of redemption data was used in the calculation of means and SDs during each study period (T1: 12,032 mo; T2: 4287 mo; T3: 6121 mo).

4 Estimate (95% confidence interval) for USD redeemed of specified category of fruits was determined for T2 (35 USD/mo) and T3 (24 USD/mo) compared with T1 (9 USD/mo) in generalized estimating equations linear regression models adjusted for child race/ethnicity, sex, and age; household food insecurity and the number of household members under age 18; and calendar month (linear and quadratic). Models also accommodated clustering of monthly observations within participating children and families.

Dollar amount redeemed in summary categories of produce type (Table 4) increased significantly for fresh fruits at T2 (10.62 USD, 95% CI: 10.16, 11.07) and T3 (7.27 USD, 95% CI: 6.82, 7.72) compared with T1; increased significantly for fresh vegetables at T2 (9.13 USD, 95% CI: 8.71, 9.54) and T3 (6.94 USD, 95% CI: 6.52, 7.35) compared with T1; increased significantly for fresh FVs (undetermined) at T2 (1.78 USD, 95% CI: 1.42, 2.13) and T3 (1.18 USD, 95% CI: 0.83, 1.54) compared with T1; and increased significantly for other FVs at T2 (7.38 USD, 95% CI: 6.85, 7.91) and T3 (4.17 USD, 95% CI: 3.63, 4.70) compared with T1.

**TABLE 4:** Dollars redeemed and percent of total CVB redemption that occurred for fruits, vegetables, and diversity^1^ of fruits, vegetables and total produce redemptions among WIC-participating households in Southern California before and during the CVB augmentation (June 2020–June 2022)

Outcome	Redemption summary measure, mean (SD)^2^			Association with CVB period^3^	
	T1	T2	T3	T2 vs. T1	T3 vs. T1
Total dollars redeemed:					
Fresh fruit	4.04 (3.94)	14.80 (11.49)	11.29 (9.97)	10.62 (10.16, 11.07)	7.27 (6.82, 7.72)
Fresh vegetables	3.64 (3.79)	12.58 (10.20)	10.56 (9.11)	9.13 (8.71, 9.54)	6.94 (6.52, 7.35)
Fresh, undetermined fruit or vegetable^4^	1.27 (3.51)	3.07 (8.88)	2.29 (7.28)	1.78 (1.42, 2.13)	1.18 (0.83, 1.54)
Other fruit or vegetable^4^	4.17 (7.81)	11.99 (13.24)	8.78 (11.34)	7.38 (6.85, 7.91)	4.17 (3.63, 4.70)
Percent of CVB redemption:					
Fresh fruit	34.34 (31.51)	35.38 (22.81)	34.79 (24.35)	0.75 (-0.29, 1.80)	0.71 (-0.43, 1.85)
Fresh vegetables	30.27 (29.68)	30.17 (21.19)	33.46 (24.09)	1.06 (0.11, 2.02)	3.09 (2.03, 4.16)
Fresh, undetermined fruit or vegetable^4^	11.06 (29.22)	7.81 (22.23)	7.14 (21.05)	-3.22 (-4.18, -2.26)	-2.82 (-3.97, -1.68)
Other fruit or vegetable^4^	24.32 (28.86)	26.62 (24.05)	24.61 (24.24)	1.50 (0.49, 2.52)	-0.78 (-1.82, 0.26)
Diversity^1^ of CVB redemption:					
Fresh fruits	5.24 (7.51)	21.97 (17.70)	14.23 (14.04)	16.32 (15.64, 17.00)	9.23 (8.65, 9.82)
Fresh vegetables	7.94 (11.29)	26.91 (20.83)	21.20 (18.69)	19.71 (18.90, 20.52)	13.53 (12.75, 14.31)
Fresh produce diversity	18.51 (15.40)	48.40 (25.91)	39.25 (22.24)	30.25 (29.27, 31.22)	21.28 (20.32, 22.23)
Fresh fruit (%) of fresh FVs redeemed	50.25 (23.65)	53.12 (21.08)	50.84 (22.45)	1.88 (0.90, 2.85)	0.38 (-0.63, 1.40)

Abbreviations: CVB, cash value benefit; FV, fruit and vegetable; PLU, price look-up code; USD, United States dollars; WIC, Special Supplemental Nutrition Program for Women, Infants and Children.

1 Diversity was captured as the sum (across all 24 fruit categories, across all 30 vegetable categories, and across all 54 produce categories for fruit, vegetable, and total diversity, respectively) of the inverse of the absolute value of 0.1538 minus the proportion of redemption (of fruits, vegetables, or all produce, respectively) in each of the individual categories of produce plus 0.1 (ie, 1/(Absolute value (0.1538-proportion in specific category)+0.1)). Higher values indicate greater diversity of fruits, vegetables, and total produce redeemed.

2 Dollar amount, percent, diversity, and fruits as a percent of total redemption are expressed as the mean (SD) amount of CVB redeemed per family for fruits, vegetables, or both during months in periods for the 3 CVB amounts issued (T1: 9 USD/mo; T2: 35 USD/mo; T3: 24 USD/mo) during the study period. Percentages of FVs do not add to 100 due to redemption via universal product codes or vendor assigned PLU which cannot be assigned to fruits or vegetables. Every observed month of redemption data was used in the calculation of means and SDs during each study period (T1: 12,032 mo; T2:4287 mo; T3: 6121 mo).

3 Estimate (95% confidence interval) for each redemption outcome was determined for T2 (35 USD/mo) and T3 (24 USD/mo) compared to T1 (9 USD/mo) in generalized estimating equations linear regression models adjusted for child race/ethnicity, sex, and age; household food insecurity and the number of household members under age 18; and calendar month (linear and quadratic). Models also accommodated clustering of monthly observations within participating children and families.

4 “Fresh, undetermined fruit or vegetable” refers to all PLU codes that cannot distinguish between a fruit or vegetable. “Other fruit or vegetable” refers to the remaining dollar amount or percent of CVB redemption that was redeemed for produce without a PLU.

At T1, fresh fruits on average accounted for the largest percentage of CVB redemption (34.34%), followed by fresh vegetables (24.32%), other FVs (eg canned or frozen; 24.32%) and fresh FVs (undetermined) (11.06%). The percent of CVB redeemed on fresh vegetables increased at T2 (1.06%, 95% CI: 0.11, 2.02) and T3 (3.09%, 95% CI: 2.03, 4.16) compared with T1; the percent redeemed on fresh FVs (undetermined) decreased at T2 (-3.22%, 95% CI: -4.18, -2.26) and T3 (-2.82, 95% CI: -3.97, -1.68) compared with T1; and the percent redeemed on other FVs increased at T2 (1.50%, 95% CI: 0.49, 2.52) compared with T1. The percent of fresh FVs redeemed as fresh fruit increased significantly at T2 (1.88%, 95% CI: 0.90, 2.85) compared with T1. Diversity scores increased significantly for fresh fruits at T2 (16.32 points, 95% CI: 15.64, 17.00) and T3 (9.23 points, 95% CI: 8.65, 9.82) compared with T1; increased significantly for fresh vegetables at T2 (19.71 points, 95% CI: 18.90, 20.52) and T3 (13.53 points, 95% CI: 12.75, 14.31) compared with T1; and increased significantly for all fresh produce at T2 (30.25 points, 95% CI: 29.27, 31.22) and T3 (21.28 points, 95% CI: 20.32, 22.23) compared with T1.

## Discussion

The increase of the WIC CVB beginning in June 2021 was associated with significant increases in the dollar amount redeemed for fresh fruits, fresh vegetables, fresh FVs (undetermined), and other FVs; significant increases for the diversity of redeemed fruits, vegetables, and all fresh produce; and a significant increase for the proportion of total CVB redemption accounted for by fresh fruit at the highest CVB amount among WIC-participating households in Los Angeles County, California. The increased amount and diversity of redemption was observed in both the monthly prevalence of any redemption and monthly dollar amount redeemed, with significant increases observed for 53 of 54 commodity groups of FVs following the introduction of the increased CVB.

The most commonly redeemed fruits in this sample across the full study period were bananas, apples, grapes, limes, and melons, similar to findings of a prior study, using 2 to 5 y old children included in the National Health and Nutrition Examination Survey (NHANES) 2009 to 2010, in which the whole fruits that contributed the most to child fruit intake were apples, bananas, grapes, and oranges [39]. Similar results were found in the national WIC Infant and Toddler Feeding Practices Study-2 (ITFPS-2) for children at age 24, 36, and 48 mo, where bananas, apples, grapes, oranges, and strawberries were the most frequently consumed fruits [40,41]. The most commonly redeemed vegetables in this study were tomatoes, onions, cucumbers, peppers, and avocados, with tomatoes and cucumbers aligning with the NHANES findings of the relative contribution of specific vegetables to child vegetable intake [39]. Results differed substantially from WIC ITFPS-2 for children at 24, 36, and 48 mo, where the most prevalent vegetables varied between tomatoes, potatoes, carrots, green beans, broccoli, and corn [40,41]. Differences between the present study and NHANES may be explained by the high prevalence of Hispanic ethnicity in the WIC-participating sample (75% compared with 39%) or in the difference in outcomes (redemption as opposed to child intake) [39].

Increases in the prevalence of any redemption were moderate to high for nearly all commodity groups, and increases in the amount (USD) redeemed following the introduction of the augmented CVB were generally greater for foods with higher baseline redemption. This led to relatively few significant changes in the percent of PLU-based CVB redemption accounted for by each commodity group. Similarly, the ratio of fruits to vegetables redeemed varied across the study period, with a modest increase in the proportion of fresh produce redeemed as fresh fruit during the 35 USD/mo period. This stability aligns with prior data on the development of food preferences early in childhood [42], which are influenced by the dietary preferences of parents [43] and the ways parents feed their infants and children [[44], [45], [46]]. Of particular note, the percent of the CVB redeemed at T2 (35 USD/mo) for other FV, a category that would include canned and frozen FVs, was modestly higher than at T1 (9 USD/mo). This suggests that while the 26 USD/mo increase in the CVB from T1 to T2 did not substantially shift the balance of fresh fruits relative to fresh vegetables redeemed, families used the increased benefits to purchase FVs that can be stored for longer than fresh FVs. This result may indicate that families found it easier to increase redemption than intake, and they turned to storage-friendly options to reap the full value of the augmented CVB. Changes to the amount of the WIC-issued CVB may not alter established preferences absent further educational interventions to promote increased intakes of less familiar types of FVs [43,47].

This study identified significant associations between the augmented CVB in T2 (35 USD/mo) and T3 (24 USD/mo) and increased diversity of fruits, vegetables, and total produce redeemed compared with T1 (9 USD/mo). This finding of higher diversity in periods during which greater values of the CVB were issued and redeemed (ie, stronger associations with diversity of redemption during T2 than T3) aligns with the limited literature on diversity of grocery purchases, with families with more limited resources generally purchasing less varied and less healthy foods [[48], [49], [50]]. Increased diversity of redemption, a proxy for household access to diverse FVs, which is known to be associated with intake [15], may contribute to more diversity in child FV intake as has been previously reported by caregivers in this sample [33]. This would also suggest that greater and more diverse redemption of FVs may be associated with improvement in child FV intake as has been suggested in this sample and in a multistate study of the WIC CVB augment [33,34]. Further research evaluating the relationship between FV redemption diversity and FV intake is needed. Another prior study using NHANES data identified that children with greater FV intake diversity consumed better quality diets with respect to FV intake [9], and early FV diversity has been associated with higher FV acceptance and intake later in childhood [51].

Associations between the CVB period and the amount (USD) and diversity of redemption were stronger during T2 (35 USD/mo) than T3 (24 USD/mo). This aligns with a prior multinational study that found differences between FV intake amounts between nations of different average income (countries of low-income, low-middle income, middle-income, high-income) and found that the consumption of FVs decreased as the relative cost of FVs (compared to income) increased [52]. Greater CVB dollar amounts may have contributed to increased FV redemption amounts and diversity via reduced costs to participants [29]. This reduction of the relative dollar amount may contribute to increased diversity of redemption through reduced concern about the potential for food waste with the introduction of the child to unfamiliar or less favored FVs [[29], [30], [31], [32]] and may further reduce the perceived cost associated with repeated exposure of a child to unfamiliar FVs [29], which could contribute to increased acceptance of diverse FVs [10,53].

This study has a number of strengths, including a large and well-characterized sample, PLU data on redemption of the CVB to allow detailed assessment of the amount of each FV and the overall diversity of FVs redeemed, and multiple months of redemption data for each family within each of the CVB periods to account for items that may be sporadically or seasonally redeemed. The study was able to accommodate adjustment for calendar month (linear and quadratic) to control for differences in seasonal availability of each individual FV commodity group. The data also have limitations, including the observational study design precluding causal inference, and the potential for residual confounding by unmeasured household sociodemographic characteristics, health behaviors, and environmental variables such as the availability of FVs at participant-accessible WIC-approved vendors. The timing of the study, during the COVID-19 pandemic, may influence results because intermittent shortages of preferred produce at WIC-approved vendors could influence CVB redemption. Additionally, redemption may be a good proxy for household availability of each FV, but data are not available to establish a relationship between redemption and consumption of the specific FV. Information about FVs purchased with other sources of funding (eg, with personal funds or with Supplemental Nutrition Assistance Program [SNAP] benefits) were unavailable for inclusion in the analyses, so inferences about the relationship between the CVB and total household purchases of FVs are not possible. All data were collected during the COVID-19 pandemic in a single urban county in Southern California and from a sample that was uniformly lower-income and predominantly Hispanic. The location of the study in Los Angeles County may lead to greater diversity of FVs redeemed (at the population level) due to a greater density of approved vendors and diverse cultural preferences in the general population. Results may not generalize to other alterations of WIC food packages, to modifications of nutrition assistance benefits provided through other programs, to changes during different time periods, or to populations that are not low-income, urban, and predominantly Hispanic.

In conclusion, the results of this study demonstrate that the increased WIC CVB was well received by participating households and allowed families to redeem greater amounts of various FVs. Given previously reported associations between limited household resources, less healthy foods purchased at vendors [48,54], greater reliance on more limited selection of foods to feed children [49,50], and trepidation about introducing novel foods due to concerns about food waste [55], the increased diversity of FVs redeemed following WIC’s introduction of the augmented CVB should be considered a success in increasing access to sufficient amounts and diversity of FVs among low-income households. Further research is needed to determine whether the enhanced CVB was associated with increases in total household purchases of FVs across all sources of funding (including WIC and SNAP benefits, personal resources), whether changes in the diversity of WIC CVB redemption observed for fresh produce was also observed for canned and frozen produce, and to determine whether the observed increase in the diversity of FV redemption with the CVB is associated with increased diversity of FV intake among WIC-participating children.

## Funding

This work was supported through awards from Healthy Eating Research, a program of the Robert Wood Johnson Foundation, and the David and Lucile Packard Foundation. The funders had no role in the design or conduct of the study, analysis of the data, drafting of the manuscript, or the decision to submit for publication.

## Author contributions

The authors’ responsibilities were as follows—SEW, LDR, LEA: designed the research; SEW, LDR, LEA, CEA, CEY conducted the research; CEA, CEY; performed the statistical analysis; CEA: wrote the initial draft of the manuscript; SEW, LDR, LEA, CEY, MMT: critically revised the manuscript; CEA: had primary responsibility for the final content; and all authors: read and approved the final manuscript.

## Conflict of interest

The authors report no conflicts of interest.

## Data availability

The data described in the manuscript will not be made available because the data are confidential administrative data of the WIC program. The code book and analytic code will be made available upon request.

## Appendix A Supplementary data

Multimedia component1 Multimedia component1
