# Supplementary material for: Increased WIC Cash Value Benefit is Associated with Greater Amount and Diversity of Redeemed Fruits and Vegetables among Participating Households
Source: Curr Dev Nutr. 2023 Aug 3;7(9):101986. doi: 10.1016/j.cdnut.2023.101986 (PMC10469066; doi:10.1016/j.cdnut.2023.101986)
Supplement: Multimedia component1 [file mmc1.docx]

**Supplemental Table 1.** Price look-up codes in each of the evaluated commodity groups for WIC Cash Value Benefit redemption (June 2020-June 2022).^a^

| **Commodity group (study)** | **Commodity group (International Federation for Produce Standards)** | **Price look-up code (specific variety, if any)** |
| --- | --- | --- |
| Apples | APPLES | 3000 (Alkmene), 3001 (Aurora/Southern Rose), 3002 (Cantared), 3003 (D'Estivale), 3004 (Discovery), 3005 (Golden Delicious Blush), 3006 (Ingrid Marie), 3007 (Lochbuie), 3008 (Rubinette), 3009 (Russet), 3010 (Cripps Red), 3011 (Worcester), 3065 (Cameo), 3066 (Cameo), 3067 (Swiss Gourmet), 3068 (Swiss Gourmet), 3069 (Gravenstein, Red), 3070 (Gravenstein, Red), 3071 (Granny Smith, Red), 3072 (Lady), 3073 (Macoun), 3074 (Greening (RI)), 3075 (Baldwin), 3076 (Melrose), 3077 (Northern Spy), 3078 (Liberty), 3104 (RoHo 3615), 3271 (Virginia Gold), 3272 (Sommerfeld), 3283 (Honeycrisp), 3284 (Red Delicious), 3285 (Golden Delicious), 3290 (Aurora/Southern Rose), 3291 (Boskoop/Belle de Boskoop), 3292 (Boskoop/Belle de Boskoop), 3293 (Scifresh), 3294 (Scifresh), 3295 (Sciearly), 3296 (Sciearly), 3297 (Scired), 3298 (Redfield), 3299 (Sonya), 3300 (Sonya), 3301 (Cripps Red), 3315 (Scilate), 3339 (Belchard - Chantecler), 3340 (Bertanne/Golden Russet), 3341 (Charles Ross), 3342 (Delblush), 3343 (Dessert), 3344 (Gloster), 3345 (Gloster), 3346 (Holstein), 3347 (Laxtons Fortune), 3348 (Lord Lambourne), 3349 (Michaelmas Red), 3350 (Reine des Reinettes/King of the Pippins), 3351 (Reine des Reinettes/King of the Pippins), 3352 (Reinettes and Heritage varieties - (incl Canada blanc Reinette du Mans, Armorique/du Vigan/Calville), 3353 (St Edmunds Pippin), 3435 (Pinova), 3438 (Ambrosia), 3442 (New York 1), 3443 (New York 2), 3444 (Green Dragon), 3445 (DS 3), 3447 (DS 22), 3460 (Red Jonaprince), 3461 (Lady Williams), 3467 (Regal 13-82), 3468 (Honeycrisp), 3484 (Dalinette), 3486 (CN121), 3487 (MN 55), 3490 (MAIA 1), 3507 (WA 38), 3510 (Ambrosia), 3511 (WA 2), 3513 (Shinano Gold), 3514 (Fengapi), 3515 (PremA 153), 3516 (PremA 153), 3519 (R10-45), 3521 (Regal D5-100), 3523 (SQ 159), 3525 (CN121), 3526 (Howell TC2), 3527 (Howell TC3), 3528 (Regal D17-121), 3529 (PremA 129), 3537 (MAIA-L), 3538 (Ipador), 3539 (IFORED Y Series), 3540 (IFORED R Series), 3541 (CIVM49), 3542 (Regal D27-16), 3600 (Antares), 3601 (Huaguan), 3602 (Belgica), 3603 (Minneiska), 3604 (Emmons), 3605 (Nicoter), 3607 (Mariri Red), 3608 (Sciros), 3612 (Nicogreen), 3613 (Fuji Brak), 3615 (Civni), 3616 (Scilate), 3618 (Opal), 3619 (Milwa), 3620 (Plumac), 3625 (Minnewashta), 3627 (PremA17), 3628 (PremA280), 3629 (CIVG198), 3630 (Co-op 43), 4015 (Red Delicious), 4016 (Red Delicious), 4017 (Granny Smith), 4018 (Granny Smith), 4019 (McIntosh), 4020 (Golden Delicious), 4021 (Golden Delicious), 4057 (Haralson), 4058 (Haralson), 4096 (Ginger Gold), 4097 (Ginger Gold), 4098 (Akane), 4099 (Akane), 4100 (Fireside), 4101 (Braeburn), 4102 (Fireside), 4103 (Braeburn), 4104 (Cortland), 4105 (Cox Orange Pippin), 4106 (Cortland), 4107 (Crab), 4108 (Crispin/Mutsu), 4109 (Crispin/Mutsu), 4110 (Crispin/Mutsu), 4111 (Crispin/Mutsu), 4112 (Regent), 4113 (Criterion), 4114 (Regent), 4115 (Criterion), 4116 (Early), 4117 (Early), 4118 (Early), 4119 (Early), 4120 (Fiesta), 4121 (Elstar), 4122 (Sciros), 4123 (Elstar), 4124 (Empire), 4125 (Empire), 4126 (Empire), 4127 (Empire), 4128 (Cripps Pink), 4129 (Fuji), 4130 (Cripps Pink), 4131 (Fuji), 4132 (Gala), 4133 (Gala), 4134 (Gala), 4135 (Gala), 4136 (Golden Delicious), 4137 (Golden Delicious), 4138 (Granny Smith), 4139 (Granny Smith), 4140 (Idared), 4141 (Jonamac), 4142 (Idared), 4143 (Jonamac), 4144 (Jonagold), 4145 (Jonagold), 4146 (Jonagold), 4147 (Jonagold), 4148 (Jonathan), 4149 (Jonathan), 4150 (Jonathan), 4151 (Jonathan), 4152 (McIntosh), 4153 (McIntosh), 4154 (McIntosh), 4155 (Paulared), 4156 (Gravenstein), 4157 (Paulared), 4158 (Gravenstein), 4160 (Pippin), 4162 (Pippin), 4167 (Red Delicious), 4168 (Red Delicious), 4169 (Rome), 4170 (Rome), 4171 (Rome), 4172 (Rome), 4173 (Royal Gala), 4174 (Royal Gala), 4176 (Southern Snap), 4177 (Spartan), 4178 (Spartan), 4179 (Spartan), 4180 (Spartan), 4181 (Stayman), 4182 (Sturmer Pippin), 4183 (Stayman), 4185 (York), 4187 (York), 4189 (Winesap), 4190 (Winesap), 4191 (Winesap), 4192 (Winesap), 4193 (RA), 4194 (RA), 4195 (RA), 4196 (RA), 4197 (RA), 4198 (RA), 4199 (RA), 4200 (RA), 4201 (RA), 4202 (RA), 4203 (RA), 4204 (RA), 4205 (RA), 4206 (RA), 4207 (RA), 4208 (RA), 4209 (RA), 4210 (RA), 4211 (RA), 4212 (RA), 4213 (RA), 4214 (RA), 4215 (RA), 4216 (RA), 4217 (RA) |
| Asparagus | ASPARAGUS | 3079 (Purple), 3392 (Green - Bunch), 3393 (White - Bunch), 3394 (Purple - Bunch), 4080 (Green), 4521 (Green), 4522 (White), 4523 (White), 4524 (Tips), 4525 (RA), 4526 (RA) |
| Avocado | AVOCADOS | 3080 (Pinkerton), 3354 (Ripe/Ready-to-Eat), 3509 (GEM), 4046 (Hass), 4221 (Green), 4222 (Green), 4223 (Green), 4224 (Green), 4225 (Hass), 4226 (Cocktail/Seedless), 4227 (RA), 4228 (RA), 4770 (Hass), 4771 (Green) |
| Banana | BANANAS | 3287 (Hawaiian plantain), 4011 (Yellow (includes Cavendish)), 4186 (Yellow (includes Cavendish)), 4229 (Burro), 4230 (Dominique), 4231 (Green), 4232 (Leaves), 4233 (Apple/Manzano), 4234 (baby/Nino), 4235 (Plantain/Macho), 4236 (Red), 4237 (RA), 4238 (RA) |
| Beans | BEANS | 3048 (Helda/Flat), 3049 (Fine), 4066 (Green/French), 4527 (Chinese Long/Snake), 4528 (Fava/Broad), 4529 (Lima), 4530 (Pole/Runner/Stick), 4531 (Purple Hull), 4532 (Shell), 4533 (Wax/Yellow), 4534 (Winged), 4535 (RA) |
| Beets | BEETS | 3273 (Golden), 4537 (Baby Golden), 4538 (Baby Red), 4539 (Bunch), 4540 (Loose), 4541 (RA) |
| Blackberries | BERRIES | 4239 (Blackberries) |
| Blueberries | BERRIES | 4240 (Blueberries) |
| Broccoli | BROCCOLI | 3082 (Crowns), 3277 (Baby), 4060 , 4547 (Broccoli Rabe (Italian Rapini)/ Chinese Broccoli (GAI LAN)), 4548 (Florettes), 4549 (RA) |
| Brussels sprouts | BRUSSELS SPROUTS | 3083 (Stalk), 4550 , 4551 (RA) |
| Cabbage | CABBAGE | 3050 (Dutch White/Winter White), 3051 (Spring Cabbage/Spring Greens), 3166 (Tuscan), 3396 (Savoy, Red), 3397 (Summer Cabbage - Pointed type), 4069 (Green), 4552 (Chinese/Napa/Wong Bok), 4554 (Red), 4555 (Savoy, Green), 4556 (RA), 4557 (RA) |
| Carrots | CARROTS | 3424 (Purple/Red - Beta Sweet), 4094 (Bunch), 4560 (Baby), 4561 (French), 4562 (Loose), 4563 (Carrot Sticks), 4564 (RA), 4565 (RA) |
| Cauliflower | CAULIFLOWER | 3320 (Romanesco/Broccoflower/Caulibroc), 3436 (Orange), 4079 , 4566 (Florettes), 4567 (Green), 4568 (Purple), 4569 (RA), 4570 (RA), 4571 (RA), 4572 , 4573 (Baby) |
| Celery | CELERY | 4070 (Bunch), 4071 (Bunch), 4575 (Hearts), 4576 (Celery Sticks), 4577 (RA), 4578 (RA), 4579 (RA), 4580 (RA), 4581 (RA), 4582 (Bunch), 4583 (Bunch) |
| Chard | CHARD (SWISS CHARD)/SILVERBEET | 4586 (Green), 4587 (Red), 4588 (RA) |
| Cherries | CHERRIES | 3357 (Regular/Red/Black), 3358 (Regular/Red/Black), 3448 (Tip Top), 4045 (Regular/Red/Black), 4258 (Golden/Rainier/White), 4259 (RA) |
| Corn | CORN | 3085 (Indian, Decorative), 3086 (Indian. Decorative), 3087 (Indian, Strawberry), 4077 (Sweet Corn, White), 4078 (Sweet Corn, Yellow), 4589 (Sweet Corn, Baby), 4590 (Sweet Corn, Bi-Color), 4591 (RA) |
| Cucumbers | CUCUMBER | 4062 (Green/Ridge/Short), 4592 (Armenian), 4593 (English/Hot House/Long Seedless/Telegraph/Continental), 4594 (Japanese/White), 4595 (Lemon), 4596 (Pickling/Gherkin), 4597 (RA) |
| Dragon fruit | PITAHAYA | 3040 (Red (skin color)), 3319 (Yellow (skin color)) |
| Fruit or vegetable (unknown) | FOR USE WITH ALL COMMODITIES | 3170 (RA), 3171 (RA), 3172 (RA), 3173 (RA), 3174 (RA), 3175 (RA), 3176 (RA), 3177 (RA), 3178 (RA), 3179 (RA), 3180 (RA), 3181 (RA), 3182 (RA), 3183 (RA), 3184 (RA), 3185 (RA), 3186 (RA), 3187 (RA), 3188 (RA), 3189 (RA), 3190 (RA), 3191 (RA), 3192 (RA), 3193 (RA), 3194 (RA), 3195 (RA), 3196 (RA), 3197 (RA), 3198 (RA), 3199 (RA), 3200 (RA), 3201 (RA), 3202 (RA), 3203 (RA), 3204 (RA), 3205 (RA), 3206 (RA), 3207 (RA), 3208 (RA), 3209 (RA), 3210 (RA), 3211 (RA), 3212 (RA), 3213 (RA), 3214 (RA), 3215 (RA), 3216 (RA), 3217 (RA), 3218 (RA), 3219 (RA), 3220 (RA), 3221 (RA), 3222 (RA), 3223 (RA), 3224 (RA), 3225 (RA), 3226 (RA), 3227 (RA), 3228 (RA), 3229 (RA), 3230 (RA), 3231 (RA), 3232 (RA), 3233 (RA), 3234 (RA), 3235 (RA), 3236 (RA), 3237 (RA), 3238 (RA), 3239 (RA), 3240 (RA), 3241 (RA), 3242 (RA), 3243 (RA), 3244 (RA), 3245 (RA), 3246 (RA), 3247 (RA), 3248 (RA), 3249 (RA), 3250 (RA), 3251 (RA), 3252 (RA), 3253 (RA), 3254 (RA), 3255 (RA), 3256 (RA), 3257 (RA), 3258 (RA), 3259 (RA), 3260 (RA), 3261 (RA), 3262 (RA), 3263 (RA), 3264 (RA), 3265 (RA), 3266 (RA), 3267 (RA), 3268 (RA), 3269 (RA), 3270 (RA), 4460 (RA), 4461 (RA), 4462 (RA), 4463 (RA), 4464 (RA), 4465 (RA), 4466 (RA),4467 (RA),4468 (RA),4469 (RA) |
| Garlic | GARLIC | 3052 (String), 3399 (Regular - Fresh/Semi-dried with leaves attached), 3400 (Regular - Smoked), 3401 (One-clove types), 4608 (Regular), 4609 (Elephant), 4610 (RA), 4611 (RA) |
| Ginger root | GINGER ROOT | 4612 (Regular), 4613 (RA) |
| Grapes | GRAPES | 3043 (Italia, Seeded), 3093 (RA), 3094 (RA), 3359 (Chasselas), 3360 (Muscat de Hambourg), 3449 (Sugrathirteen), 3450 (Sugranineteen), 3451 (Sugrathirtyfour), 3452 (Sugrathirtyfive), 3469 (Sugrasixteen), 3491 (ARRA FIFTEEN), 3492 (ARRA TWENTYNINE), 3496 (IFG Core Red Seedless), 3497 (IFG Core Black Seedless ), 3498 (IFG Core Green Seedless), 3499 (IFG Novelty Red Seedless), 3500 (IFG Novelty Black Seedless), 3501 (IFG Novelty Green Seedless), 3502 (ARRA TWENTYSEVEN), 3503 (ARRA TWENTYEIGHT), 3504 (ARRA THIRTY), 3505 (ARRA THIRTYTWO), 3506 (Sweet Scarlet), 3508 (Thomcord), 3530 (Jupiter), 3531 (Sugra Family Black Seedless), 3532 (Sugra Family Green Seedless), 3533 (Sugra Family Novelty Seedless), 3534 (Sugra Family Red Seedless), 4022 (White/Green Seedless - Peerlette Seedless/Thompson Seedless), 4023 (Red Seedless - Flame/Ruby/Emperatriz), 4056 (Blue/Black Seedless (All other varieties not listed above including Beauty and Autumn Royal)), 4270 (Blue/Black Seeded (Ribier/Exotic/Niabel)), 4271 (Champagne), 4272 (Concord), 4273 (Red Seeded (Cardinal/Emperor/Queen/Christmas Rose)), 4274 (White/Green Seeded (All others not listed)), 4275 (RA), 4276 (RA), 4277 (RA), 4278 (RA), 4497 (Sugraone), 4498 (White/Green Seedless (All others not listed above (including Autumn King))), 4499 (Crimson/Majestic), 4635 (Red Seedless (All others not listed under red seedless)), 4636 (Red Globe), 4637 (Red Seeded (All others not listed above)), 4638 (Fantasy/Marroo), 4957 (Blue/Black Seeded (All others not listed above)) |
| Kale | KALE | 3095 (Multicolor), 4627 |
| Kiwifruit | KIWIFRUIT | 3279 (Golden), 3280 (Regular), 3517 (RA - Gold Derivative Varieties), 4030 (Regular), 4301 (RA) |
| Leafy green | BELGIAN ENDIVE (WITLOOF CHICORY) | 3395 (Red), 4543 |
|  | BOK CHOY (PAK CHOI) | 3163 (Shanghai), 4544 (Small/Baby), 4545 |
|  | CACTUS LEAVES (Nopales/Cactus Pads) | 3471 (Baby), 4558 |
|  | CHIPILIN LEAF | 3479 |
|  | CHOY SUM/PAK CHOI SUM | 3322 , 3323 (Baby) |
|  | ESCAROLE/BATAVIAN CHICORY | 3324 (Red), 4605 (Green) |
|  | FIDDLEHEAD FERNS | 4606 |
|  | GREENS | 4614 (Collard), 4615 (Dandelion), 4616 (Mustard (Synonymous with Gai (Gui) Choy)), 4617 (Polk Greens), 4618 (Texas Mustard), 4619 (Turnip), 4620 (RA), 4621 (RA), 4622 (RA), 4623 (RA), 4624 (RA) |
|  | QUELITES | 3478 |
|  | RADICCHIO | 3165 (Treviso), 3168 (Castlefranco), 4738 |
|  | WATERCRESS | 4815 |
| Lemons | LEMONS | 3362 (Without postharvest treatment), 3617 (Seedless), 3626 (Meyer), 4033 , 4053 , 4304 (RA), 4958 |
| Lettuce | LETTUCE | 3097 (Romaine, Red), 3098 (Boston, Red), 3169 (Catalogna), 3325 (Lollo Bionda/Coral - Green), 3326 (Lollo Rossa/Coral - Red), 3327 (Mignonette (Compact red-tinged butterhead varieties)), 3328 (Mixed small-leaf salad (eg Sucrine, Mesclun, Rocket/Arugula)), 3329 (Oak Leaf - Green), 3330 (Oak Leaf - Reg), 4061 (Iceberg), 4075 (Red Leaf), 4076 (Green Leaf), 4631 (Bibb/Flat/Round), 4632 (Boston/Butter), 4633 (Hydroponic), 4634 (Iceberg), 4639 (Mache), 4640 (Romaine/Cos), 4641 (RA), 4642 (RA), 4643 (RA) |
| Limes | LIMES | 4048 (Regular (incl. Persian, Tahiti & Bearss)), 4305 (Key (incl. Mexican & West Indian)), 4306 (RA) |
| Mango | MANGO | 3114 (Green), 3363 (Kensington Pride), 3364 (R2E2), 3365 (Ripe/Ready-to-Eat), 3464 (B-74), 3488 (Red), 3621 (Francis), 4051 (Red (Includes Tommy Atkins, Kent, Palmer, Vandyke, Edward,Hayden)), 4311 (Green (Includes Keitt and Francis)), 4312 (Yellow (Includes Oro, Ataulfo/Honey Manila)), 4313 (RA), 4314 (RA), 4315 (RA), 4316 (RA), 4584 (Green (Includes Keitt and Francis)), 4959 (Red (Includes Tommy Atkins, Kent, Palmer Vandyke, Edward, Hayden)), 4961 (Yellow) |
| Melon | MELON | 3033 (Charentais), 3034 (Charentais), 3100 (Gold Honeydew), 3101 (Piel de Sapo), 3281 (Watermelon - Orange, Seedless), 3289 (Sprite), 3306 (Charentais), 3307 (Charentais), 3308 (Watermelon - Orange, Seedless), 3367 (Glasshouse - Netted varieties), 3368 (Ogen), 3421 (Watermelon - Mini Seedless), 3456 (Winter), 3470 (Watermelon - Red, Small Seeds), 3494 (Watermelon - Yellow Mini Seedless), 3622 (Honey Green), 3623 (Hami), 3624 (Korean), 4031 (Watermelon - Red ), 4032 (Watermelon - Red, Seedless), 4034 (Honeydew/White Honeydew), 4049 (Cantaloupe/Rockmelon), 4050 (Cantaloupe/Rockmelon), 4317 (Canary/Yellow Honeydew), 4318 (Cantaloupe/Muskmelon), 4319 (Cantaloupe/Muskmelon), 4320 (Casaba), 4321 (Cinnabar), 4322 (Crenshaw), 4324 (French Afternoon), 4325 (French Breakfast), 4326 (Galia), 4327 (Orange Flesh/Cantaline), 4329 (Honeydew/White Honeydew), 4330 (Mayan), 4331 (Mickey Lee Watermelon/Sugarbaby), 4332 (Muskmelon), 4333 (Pepino), 4334 (Persian), 4335 (Prince), 4336 (Santa Claus), 4337 (Saticoy), 4338 (Sharlin), 4339 (Spanish/Tendral), 4340 (Watermelon - Yellow), 4341 (Watermelon - Yellow, Seedless), 4342 (RA (includes pre-cut melons)), 4343 (RA (includes pre-cut melons)), 4344 (RA (includes pre-cut melons)), 4345 (RA (includes pre-cut melons)), 4346 (RA (includes pre-cut melons)), 4347 (RA (includes pre-cut melons)), 4348 (RA (includes pre-cut melons)), 4349 (RA (includes pre-cut melons)), 4350 (RA (includes pre-cut melons)), 4351 (RA (includes pre-cut melons)), 4352 (RA (includes pre-cut melons)), 4353 (RA (includes pre-cut melons)), 4354 (RA (includes pre-cut melons)), 4355 (RA (includes pre-cut melons)), 4356 (RA (includes pre-cut melons)), 4357 (RA (includes pre-cut melons)), 4358 (RA (includes pre-cut melons)), 4359 (RA (includes pre-cut melons)), 4360 (RA (includes pre-cut melons)), 4361 (RA (includes pre-cut melons)), 4362 (RA (includes pre-cut melons)), 4363 (RA (includes pre-cut melons)), 4364 (RA (includes pre-cut melons)), 4365 (RA (includes pre-cut melons)), 4366 (RA (includes pre-cut melons)), 4367 (RA (includes pre-cut melons)), 4368 (RA (includes pre-cut melons)), 4369 (RA (includes pre-cut melons)), 4370 (RA (includes pre-cut melons)), 4371 (RA (includes pre-cut melons)), 4372 (RA (includes pre-cut melons)), 4373 (RA (includes pre-cut melons)), 4374 (RA (includes pre-cut melons)), 4375 (RA (includes pre-cut melons)), 4376 (RA (includes pre-cut melons)) |
| Mushroom | MUSHROOMS | 3102 (Morel), 3103 (Enoki), 3404 (Cep), 3405 (Fairy Ring Champignon), 3406 (Grey - Tricholoma), 3407 (Grisette), 3408 (Horn of Plenty/Black Trumpet), 3409 (Pioppino), 3410 (Saffron Milk-Cap), 3411 (Sheep Polypore), 4085 (Regular), 4645 (Regular, Button), 4646 (Black Forest), 4647 (Chanterelle), 4648 (Cremini/Brown/Swiss Brown), 4649 (Oyster), 4650 (Portabella (Synonymous with Cremini, Brown, Swiss Brown Mushrooms)), 4651 (Shiitake), 4652 (Wood Ear), 4653 (RA), 4654 (RA) |
| Nectarine | NECTARINE | 3035 (White Flesh, Tree Ripened, Ready-to-eat), 3369 (Nectavigne (Red Flesh)), 3437 (Flat Yellow), 3439 (White Flesh Flat), 4035 (Yellow Flesh), 4036 (Yellow Flesh), 4188 (White Flesh, Tree Ripened, Ready-to-eat), 4377 (Yellow Flesh, Tree Ripened, Ready-to-eat), 4378 (Yellow Flesh, Tree Ripened, Ready-to-eat), 4379 (RA), 4380 (RA) |
| Non-leafy green | ALFALFA SPROUTS | 4514 |
|  | ARTICHOKES | 3391 (Rouge Salambo (Red)), 4084 , 4516 , 4517 (Purple), 4518 (Purple), 4519 (Baby/Cocktail), 4520 (RA), 4762 |
|  | BEAN SPROUTS | 4536 (Mung Bean Sprouts) |
|  | CHIVES | 3462 (Garlic/Chinese), 4888 |
|  | GAI LAN | 3160 (Synonymous with Chinese Broccoli) |
|  | OKRA | 4655 (Regular (Green)), 4656 (Chinese), 4657 (Red) |
|  | PEAS | 4092 (Chinese Snow Pea/Pea Pod/Mange Tout), 4673 (Blackeyed), 4674 (Green), 4675 (Sugar Snap), 4676 (RA) |
| Onions | ONIONS | 3286 (Sweet red Italian - flat), 3331 (Red Fresh - Bunch), 3412 (Yellow/Brown Fresh - Bunch), 3493 (Tearless Sweet), 3520 (SK-20), 3535 (Sweetheart), 4068 (Green (Scallions)/Spring), 4082 (Red), 4093 (Yellow/Brown), 4159 (Vidalia), 4161 (Texas Sweet), 4163 (Walla Walla), 4164 (Maui), 4165 (California Sweet), 4166 (Other Sweet), 4658 (Boiling), 4659 (Bulb), 4660 (Pearl), 4661 (Pickling, White), 4662 (Shallots), 4663 (White), 4665 (Yellow/Brown), 4666 (RA), 4667 (RA), 4668 (RA), 4669 (RA), 4670 (RA) |
| Oranges | ORANGES | 3027 (Shamouti), 3028 (Delta Seedless), 3036 (Midknight), 3107 (Navel), 3108 (Valencia), 3109 (Seville (Marmalade type)), 3110 (Navel, Cara Cara (Red)), 3153 (Delta Seedless), 3154 (Delta Seedless), 3155 (Midknight), 3156 (Midknight), 3309 (Lima), 3310 (Pera), 3370 (Maltaise), 3371 (Salustiana), 3372 (Navelate (and other late Navel varieties)), 3373 (Navelina (incl. Newhall)), 3374 (Without postharvest treatment), 4012 (Navel), 4013 (Navel), 4014 (Valencia), 4014 (Valencia), 4381 (Blood), 4382 (Juice), 4384 (Navel), 4385 (Navel), 4386 (Temple), 4387 (Temple), 4388 (Valencia), 4388 (Valencia), 4389 (RA), 4390 (RA), 4391 (RA), 4392 (RA), 4393 (RA) |
| Other citrus | GRAPEFRUIT | 3092 (OroBlanco/Sweetie), 3129 (Pummelo - Red), 3152 (Melogold), 3157 (White), 3158 (White), 3159 (White), 3361 (Without p/harvest treatment), 4027 (Ruby/Red/Pink (includes Ray Ruby, Ruby, Ruby Red)), 4047 (Ruby/Red/Pink (Includes Ray Ruby, Ruby, Ruby Red)), 4279 (Pummelo - White), 4280 (Ruby/Red/Pink (Includes Ray Ruby, Ray, Ruby Red)), 4281 (Ruby/Red/Pink (ncludes Ray Ruby, Ruby, Ruby Red)), 4282 (Ruby/Red/Pink (Includes Ray Ruby, Ruby, Ruby Red)), 4283 (Ruby/Red/Pink (Includes Ray Ruby, Ray, Ruby Red)), 4284 (Deep Red), 4285 (Deep Red), 4286 (Deep Red), 4287 (Deep Red), 4288 (Deep Red), 4289 (Deep Red), 4290 (White), 4291 (White), 4292 (White), 4293 (White), 4294 (White), 4295 (White), 4296 (RA), 4297 (RA), 4298 (RA), 4491 (Ruby/Red/Pink (includes Ray Ruby, Ruby, Ruby Red)), 4492 (Ruby/Red/Pink (includes Ray Ruby, Ruby, Ruby Red)), 4493 (Ruby/Red/Pink (Includes Ray Ruby, Ruby, Ruby Red)), 4494 (Deep Red), 4495 (Deep Red), 4496 (Deep Red) |
|  | KUMQUAT | 4303 |
|  | TANGELO | 4383 (Minneola), 4456 , 4459 (Jamaican) |
| Other fruit | APRICOTS | 3044 (Black Velvet), 3302 (Regular), 3422 (InterSpecific), 3614 (Red), 4218 (Regular), 4219 (RA), 4861 (Dried) |
|  | BERRIES | 3081 (Saskatoon), 3304 (Loganberries), 4241 (Boysenberries), 4242 (Cranberries), 4243 (Gooseberries), 4252 (RA), 4253 (RA) |
|  | BREADFRUIT | 4254 |
|  | CACTUS (PRICKLY) PEAR | 4255 |
|  | CARAMBOLA (STARFRUIT) | 4256 |
|  | CHERIMOYA | 4257 |
|  | COCONUTS | 4260 (In Husk/Waternut), 4261 (Husked), 4262 (RA), |
|  | DATES | 3045 (Fresh, On Branch), 3046 (Fresh, Frozen), 3047 (Medjool), 4263 (Fresh - Regular), 4264 (Fresh - RA), 4862 (Dried), 4863 (RA - Dried) |
|  | FIGS | 3337 ((Dried Fruit)), 4266 (Black), 4267 (Brown), 4268 (White/Green), 4269 (RA) |
|  | GUAVA | 4299 |
|  | JACKFRUIT | 3454 (Green), 3455 (Yellow) |
|  | LONGAN | 4307 |
|  | LOQUATS | 4308 |
|  | LYCHEES | 4309 |
|  | MAMEY | 4310 |
|  | MANGOSTEEN | 3042 |
|  | OTHER FRUITS | 4471 (RA), 4472 (RA), 4473 (RA), 4474 (RA), 4475 (RA), 4476 (RA), 4477 (RA), 4478 (RA), 4479 (RA), 4480 (RA), 4481 (RA), 4482 (RA), 4483 (RA), 4484 (RA), 4485 (RA), 4486 (RA), 4487 (RA), 4488 (RA), 4489 (RA), 4490 (RA) |
|  | PAPAYA/PAWPAW | 3111 (Red-Fleshed (Solo Sunrise)), 3112 (Meridol), 4052 (Regular), 4394 (Regular), 4395 (Cooking/Mexican), 4396 (RA) |
|  | PASSION FRUIT | 3038 (Granadilla, Orange), 3311 (Curuba/Banana), 3312 (Granadilla - Yellow/Maracuja), 4397 (Purple), 4398 (RA) |
|  | PERSIMMON | 3459 (Shiny Red), 4427 (Regular (American Persimmon)), 4428 (Japanese/Sharonfruit (Kaki)), 4429 (RA) |
|  | PHYSALIS/CAPE GOOSEBERRY/GROUND CHERRY | 3039 |
|  | POMEGRANATE | 3127 , 3440 , 4445 , 4446 (RA) |
|  | QUINCE | 4447 |
|  | RAMBUTAN | 3041 |
|  | SOURSOP | 3381 |
|  | SUGAR APPLE | 3382 |
| Other root vegetable | CELERY ROOT/CELERIAC | 3321 (With leaves attached), 4585 |
|  | DAIKON | 4598 ((See also RADISH)) |
|  | FENNEL | 4515 (Florence/Sweet Fennel/Fennel Bulb) |
|  | HORSERADISH ROOT | 4625 |
|  | JICAMA/YAM BEAN | 4626 |
|  | KOHLRABI | 3096 (Purple/Red/All Other Colors), 4628 |
|  | LEEKS | 3402 (Regular - Bunch), 3403 (Baby - Bunch), 4629 (Regular), 4630 (Baby) |
|  | MALANGA | 4644 |
|  | PARSNIP | 3053 (Baby), 4672 |
|  | RUTABAGAS (SWEDE) | 4747 (Regular), 4748 (RA) |
|  | TARO ROOT (DASHEEN) | 4794 , 4795 |
|  | TURNIP | 4095 (Yellow), 4809 (Baby), 4810 (Bunch/Banded), 4811 (Purple Top), 4812 (White), 4813 (RA) |
|  | YUCA ROOT/CASSAVA/MANIOC | 4819 |
| Other vegetable | EGGPLANT (AUBERGINE) | 3089 (Chinese), 3090 (Thai), 4081 (Regular), 4599 (Baby), 4600 (Baby White), 4601 (Japanese), 4602 (White), 4603 (RA) |
|  | OTHER VEGETABLES | 4500 (RA), 4501 (RA), 4502 (RA), 4503 (RA), 4504 (RA), 4505 (RA), 4506 (RA), 4507 (RA), 4508 (RA), 4509 (RA), 4510 (RA), 4511 (RA), 4512 (RA), 4513 (RA), 4820 (RA), 4821 (RA), 4822 (RA), 4823 (RA), 4824 (RA), 4825 (RA), 4826 (RA), 4827 (RA), 4828 (RA), 4829 (RA), 4830 (RA), 4831 (RA), 4832 (RA), 4833 (RA), 4834 (RA), 4835 (RA), 4836 (RA), 4837 (RA), 4838 (RA), 4839 (RA), 4840 (RA), 4841 (RA), 4842 (RA), 4843 (RA), 4844 (RA), 4845 (RA), 4846 (RA), 4847 (RA), 4848 (RA), 4849 (RA), 4850 (RA), 4851 (RA), 4852 (RA), 4853 (RA), 4854 (RA), 4855 (RA), 4856 (RA), 4857 (RA), 4858 (RA), 4859 (RA) |
|  | TAMARIND | 4448 |
| Peaches | PEACHES | 3113 (Flat White Flesh (Saturn type)), 3115 (Flat Yellow Flesh), 3116 (Yellow Flesh (Tree Ripened/Ready-to-eat)), 3117 (Yellow Flesh (Tree Ripened/Ready-to-eat)), 3313 (White Flesh - Tree Ripened/ Ready-to-eat), 3314 (White Flesh - Tree Ripened/ Ready-to-eat), 3375 (de Vigne & Sanguine (Red Flesh)), 4037 (Yellow Flesh), 4038 (Yellow Flesh), 4043 (Yellow Flesh - Tree Ripened/Ready-to-eat), 4044 (Yellow Flesh - Tree Ripened/Ready-to-eat), 4399 (Indian), 4400 (White Flesh), 4401 (White Flesh), 4402 (Yellow Flesh), 4403 (Yellow Flesh), 4404 (RA), 4405 (RA) |
| Pears | PEARS | 3012 (Abate Fetel), 3013 (Beurre Hardy), 3014 (Bon Rouge), 3015 (Clara Friis), 3016 (Concorde), 3017 (Conference), 3018 (Durondeau), 3019 (Flamingo), 3020 (General Leclerc), 3021 (Guyot), 3022 (Josephine), 3023 (Passe Crassane), 3024 (Rocha), 3025 (Rosemarie), 3026 (Triumph de Vienne), 3118 (Starkrimson), 3316 (Carmen), 3317 (Angelys), 3318 (Passe Crassane), 3376 (Alexander Lucas), 3377 (Louise Bonne), 3378 (Santa Maria), 3420 (Belle du Jumet), 3434 (Tosca), 3466 (Cape Rose), 3485 (Harovin Sundown), 3489 (Cepuna), 3495 (Celina), 3518 (Oksana), 3522 (HW624), 3606 (Sweet Sensation), 4024 (Bartlett/Williams/WBC), 4025 (Anjou), 4026 (Bosc/Beurre Bosc), 4406 (Asian/Nashi - White), 4407 (Asian/Nashi - Yellow), 4408 (Asian/Nashi - Brown), 4409 (Bartlett/Williams/WBC), 4410 (Bartlett - Red/Red Sensation), 4411 (Bosc/Beurre Bosc), 4412 (Bosc/Beurre Bosc), 4413 (Bosc/Beurre Bosc), 4414 (Comice/ Doyenne du Comice), 4415 (Red), 4416 (Anjou), 4417 (Anjou - Red), 4418 (Forelle/Corella), 4419 (French), 4420 (King Royal), 4421 (Packham/Packhams Triumph), 4422 (Seckel), 4423 (Tree Ripened), 4424 (Winter Nelis/Honey), 4425 (RA), 4426 (RA), 4553 (Taylors Gold), 4890 (Chinese Yali), 4960 (Fragrant) |
| Peppers | PEPPERS (CAPSICUMS) | 3054 (Elongated (Clovis Red/Lamuyo Red)), 3055 (Elongated (Clovis Green/Lamuyo Green)), 3056 (Elongated (Clovis Yellow/Lamuyo Yellow)), 3057 (Elongated (Clovis Orange/Lamuyo Orange)), 3058 (Elongated (Clovis White/Lamuyo White)), 3119 (Bell, Greenhouse - Green), 3120 (Bell, Greenhouse - Green), 3121 (Bell, Greenhouse - Orange), 3122 (Bell, Greenhouse - White), 3123 (Bell, Greenhouse - Brown), 3124 (Bell, Greenhouse - Purple), 3125 (Habanero), 3413 (Tabasco), 3465 (Stripy Bell), 4065 (Bell, Field Grown - Green), 4088 (Bell, Field Grown - Red), 4677 (Anaheim (Green and Red)), 4678 (Banana (Yellow Long)), 4679 (Bell, Field Grown - Brown), 4680 (Bell, Field Grown - Golden (Yellow)), 4681 (Bell, Field Grown - Green), 4682 (Bell, Field Grown - Orange), 4683 (Bell, Field Grown - Purple), 4684 (Bell, Field Grown - White), 4685 (Chili - Dried), 4686 (Chili - Green), 4687 (Cubanelle), 4688 (Bell, Greenhouse - Red), 4689 (Bell, Greenhouse - Yellow), 4690 (Hot (Hungarian Hot)), 4691 (Hot Mixed), 4692 (Hungarian Wax), 4693 (Jalapeno - Green/Mexican Green), 4694 (Jalapeno - Red/Mexican Red), 4695 (Japanese - Red), 4696 (Long Hot - Green), 4697 (Long Hot - Red), 4698 (Morita Chili), 4699 (Negro), 4700 (New Mexico), 4701 (Pasilla - Green), 4702 (Pasilla - Red), 4703 (Pasilla Pod), 4704 (Pinole), 4705 (Poblano), 4706 (Red Cheese), 4707 (Red Finger), 4708 (Red Pimiento/ Red Sweet Long), 4709 (Serrano), 4710 (RA), 4711 (RA), 4712 (RA), 4713 (RA), 4714 (RA), 4715 (RA), 4716 (RA), 4717 (RA), 4718 (RA), 4719 (RA), 4720 (RA), 4721 (RA), 4722 (RA), 4772 (Chili - Yellow) |
| Pineapple | PINEAPPLE | 3037 (Queen), 3379 (Mini), 3380 (Perola), 4029 , 4430 , 4431 (Jet Fresh), 4432 (Jet Fresh), 4433 (RA), 4864 (Dried) |
| Plums | PLUMCOT (INTERSPECIFIC PLUM) | 3126 (RA), 3278 , 3609 (Red), 3610 (Green), 3611 (Black) |
| Plums | PLUMS | 3457 (President), 3536 (Sweet Pixie), 4039 (Black (Includes Ambra, Black Beaut, Prima Black, Blackamber, Black Torch, Catalina, Challenger, Black Diamond, Friar, Royal Diamond, Black Knight, Freedom, Black Flame, Howard Sun, Angeleno)), 4040 (Black (Includes Ambra, Black Beaut, Prima Black, Blackamber, Black Torch, Catalina, Challenger, Black Diamond, Friar, Royal Diamond, Black Knight, Freedom, Black Flame, Howard Sun, Angeleno)), 4041 (Red (Includes Santa Rosa, Late Santa Rosa, Red Beaut, Rich Red, Spring Beaut, First Beaut, Royal Red, Red Jewel, Rose Zee, Royal Zee, Ace, Aleta Rose, Burgandy, July Santa Rosa, Frontier, Fortune, Grand Rosa, Red Lane, Red Rosa, Casselman, Autumn Rosa, Mi), 4042 (Red (Includes Santa Rosa, Late Santa Rosa, Red Beaut, Rich Red, Spring Beaut, First Beaut, Royal Red, Red Jewel, Rose Zee, Royal Zee, Ace, Aleta Rose, Burgandy, July Santa Rosa, Frontier, Fortune, Grand Rosa, Red Lane, Red Rosa, Casselman, Autumn Rosa, Mi), 4434 (Green (Includes Dolly, Kelsey, Wickson)), 4435 (Green (Includes Dolly, Kelsey, Wickson)), 4436 (Italian Prune/Sugar), 4437 (Purple (Includes Queen Rosa, Laroda, Nublana, Queen Ann, Simka, El Dorado)), 4438 (Purple (Includes Queen Rosa, Laroda, Nublana, Queen Ann, Simka, El Dorado) ), 4439 (Tree Ripened), 4440 (Tree Ripened), 4441 (Yellow (Includes Golden Globe)), 4442 (Yellow (Includes Golden Globe)), 4443 (RA), 4444 (RA), |
| Potato | POTATO | 3128 (Purple), 3414 (Baking - White), 3415 (Baking - Red and Red Eye varieties), 4072 (Russet), 4073 (Red), 4083 (White), 4723 (Creamer - Red), 4724 (Creamer - White), 4725 (Russet), 4726 (Long - White), 4727 (Yellow), 4728 (RA), 4729 (RA), 4730 (RA), 4731 (RA), 4732 (RA), 4733 (RA) |
| Radish | RADISH | 4089 (Bunched Red), 4739 (Black), 4740 (Bunched White), 4741 (Italian Red), 4742 (Red), 4743 (White/Icicle), 4744 (RA) |
| Raspberries | BERRIES | 4054 (Raspberries - Red), 4244 (Raspberries - Black), 4245 (Raspberries - Golden) |
| Spinach | SPINACH | 3332 (Baby), 3417 (New Zealand Spinach), 4090 (Regular/Bunched), 4749 (RA) |
| Squash | BITTER MELON/BITTER GOURD, Foo Qua | 4783 (Foo Qua) |
|  | PUMPKIN | 3130 (Jumbo), 3131 (Decorative (Painted)), 3132 (White), 3133 (White - Mini), 3134 (Pie Pumpkin), 3631 (Pink), 4734 (Mini), 4735 (Regular), 4736 (RA), 4737 (RA) |
|  | SQUASH | 3059 (Crown Prince), 3060 (Vegetable Marrow), 3140 (Cucuzza), 3141 (Opo), 3142 (Carnival), 3143 (Acorn - baby), 3418 (Zucchini/Courgette - Round), 3441 (Butterkin), 4067 (Zucchini/Courgette), 4086 (Yellow Zucchini/Gold Bar/Yellow Courgette), 4750 (Acorn/Table Queen), 4751 (Acorn - Golden), 4752 (Acorn - Swan White Table Queen), 4753 (Australian Blue), 4754 (Baby Scallopini), 4755 (Baby Summer (Green)), 4756 (Baby Green Zucchini/Courgette), 4757 (Banana), 4758 (Buttercup), 4759 (Butternut), 4760 (Calabaza), 4761 (Chayote/Choko), 4763 (Delicata/Sweet Potato), 4764 ((Sweet) Dumpling), 4765 (Gem), 4766 (Golden Delicious), 4767 (Golden Nugget), 4768 (Hubbard), 4769 (Kabocha), 4773 (Patty Pan/Summer), 4774 (Red Kuri), 4775 (Scallopini), 4776 (Spaghetti/Vegetable Spaghetti), 4777 (Sunburst (Yellow)), 4779 (Sweet Mama), 4780 (Turban), 4781 (White), 4782 (Yellow - Straightneck), 4784 (Yellow - Crookneck), 4785 (RA), 4786 (RA), 4787 (RA), 4788 (RA), 4789 (RA) |
| Strawberries | BERRIES | 3355 (Strawberries - Nominal 500g/1 litre), 3356 (Strawberries - Nominal250g/1/2 litre), 4028 (Strawberries), 4246 (Strawberries), 4247 (Strawberries), 4248 (Strawberries), 4249 (Strawberries), 4250 (Strawberries), 4251 (Long-stemmed Strawberries), 4323 (Strawberries) |
| Sweet potato | BONIATO | 4546 (See also SWEET POTATO) |
|  | SWEET POTATO/YAM/KUMARA | 3288 (RA), 3333 (Red/Orangy White Flesh), 3334 (Red/Orangy White Flesh), 3474 (Saffron), 4074 (Red/Orangy Red Flesh), 4091 (White), 4816 (Golden), 4817 (Red/Orangy Red Flesh) |
| Tangerine/mandarin | TANGERINES/MANDARINS | 3029 (Satsuma), 3030 (Nova (includes Clemenvilla, Suntina)), 3031 (Jamaican Tangor (includes Ortanique, Mandor, Mandora, Tambor, Topaz, Ortanline)), 3032 (Ellendale), 3144 (Fall Glo), 3383 (Clementine), 3384 (Clementine), 3385 (Clementine), 3386 (Clementine (With leaves attached)), 3387 (Clementine (Without p/harvest treatment)), 3388 (Satsuma -Clauselina), 3389 (Satsuma), 3425 (Ellendale), 3426 (Ellendale), 3427 (Ellendale), 3428 (Honey/Murcott), 3429 (Honey/Murcott), 3430 (Honey/Murcott), 3431 (Imperial), 3432 (Imperial), 3433 (Imperial), 3524 (C37), 3632 (Dekopon), 4055 , 4449 (Sunburst), 4450 (Clementine (includes Fortune)), 4451 (Dancy), 4452 (Fairchild), 4453 (Honey/Murcott), 4454 (Kinnow), 4455 (Mandarin/Royal), 4457 (RA), 4458 (RA) |
| Tomato | TOMATOES | 3061 (Beef/Beefsteak), 3145 (Plum/Italian/Saladette/Roma (Yellow)), 3146 (Cherry - Red, On the Vine), 3147 (Cherry - Yellow, On the Vine), 3148 (Regular - Yelllow, On the Vine (Truss)), 3149 (Regular - Orange, On the Vine), 3150 (Cocktail/Intermediate - Red), 3151 (Vine Ripe, Regular - Red), 3282 (Plum/Italian/Saladette/Roma on the vine - Red), 3335 (Cocktail/Intermediate - Red/On the Vine (Truss)), 3336 (Cocktail/Intermediate - Red/Plum/Italian/Saladette/Roma/On the Vine (Truss)), 3423 (Heirloom ), 3458 (Cherry - Orange), 3512 (Round), 4063 (Regular - Red), 4064 (Regular - Red), 4087 (Plum/Italian/Saladette/Roma - Red), 4664 (Regular - Red, On the Vine (Truss)), 4778 (Regular - Yellow), 4796 (Cherry - Red), 4797 (Cherry - Yellow), 4798 (Greenhouse/Hydroponic/Regular - Red), 4799 (Greenhouse/Hydroponic/Regular - Red), 4800 (Native/Home Grown), 4801 (Tomatillos/Husk Tomatoes), 4802 (Dried), 4803 (Teardrop/Pear - Red), 4804 (Teardrop/Pear - Yellow), 4805 (Vine Ripe, Regular - Red), 4806 (RA), 4807 (RA), 4808 (RA) |

Abbreviations: RA, retailer assigned;

^a^ Price look-up codes are presented only for produce in commodity groups for which there was any redemption in the study sample. Other categories of commodities with price look-up codes (e.g. herbs and nuts) are not included as they are not purchased with WIC benefits.

**Supplemental Table 2**. Percent of PLU-based CVB redemption that occurred in categories of vegetables among WIC-participating households in Southern California before and during the CVB augmentation (June 2020-June 2022).

|  | Percent of CVB Redeemed ^a^ | | | | |
| --- | --- | --- | --- | --- | --- |
|  | T1 ^a^ | T2 ^a^ | T3 ^a^ | T2 v T1 ^b^ | T3 v T1 ^b^ |
| Asparagus | 0.5 (4.2) | 0.4 (2.5) | 0.3 (2.2) | -0.02 (-0.14, 0.10) | **-0.21 (-0.32, -0.11)** |
| Avocados | 3.9 (11.6) | 4.6 (7.8) | 4.6 (11.5) | **0.68 (0.31, 1.04)** | **0.63 (0.21, 1.05)** |
| Beans | 0.7 (4.8) | 0.6 (2.6) | 0.6 (2.9) | -0.04 (-0.19, 0.10) | -0.12 (-0.26, 0.02) |
| Beets | 0.1 (1.7) | 0.1 (1.1) | 0.1 (1.1) | 0.01 (-0.05, 0.06) | 0.01 (-0.04, 0.06) |
| Broccoli | 1.0 (5.1) | 1.0 (3.0) | 0.9 (3.2) | -0.09 (-0.24, 0.06) | -0.06 (-0.21, 0.10) |
| Brussels sprouts | 0.1 (1.2) | 0.1 (0.9) | 0.1 (1.1) | 0.03 (-0.01, 0.08) | 0.02 (-0.02, 0.05) |
| Cabbage | 0.6 (3.8) | 0.7 (2.4) | 0.8 (3.4) | -0.00 (-0.12, 0.11) | **0.19 (0.07, 0.31)** |
| Carrots | 0.6 (3.3) | 0.6 (2.0) | 0.6 (2.5) | **0.11 (0.01, 0.20)** | 0.03 (-0.07, 0.14) |
| Cauliflower | 0.5 (4.3) | 0.4 (2.6) | 0.5 (3.2) | -0.05 (-0.17, 0.08) | 0.04 (-0.07, 0.15) |
| Celery | 0.6 (4.1) | 0.5 (2.3) | 0.6 (2.9) | -0.03 (-0.15, 0.10) | -0.01 (-0.13, 0.11) |
| Chard | 0.1 (1.8) | 0.1 (1.2) | 0.1 (1.5) | -0.05 (-0.12, 0.01) | 0.00 (-0.05, 0.06) |
| Corn | 1.1 (5.7) | 1.1 (3.4) | 1.2 (4.1) | 0.04 (-0.13, 0.21) | 0.00 (-0.18, 0.18) |
| Cucumbers | 1.6 (5.8) | 1.7 (3.2) | 1.7 (3.9) | -0.02 (-0.19, 0.15) | 0.12 (-0.05, 0.29) |
| Garlic | 0.4 (3.2) | 0.5 (2.4) | 0.6 (2.8) | 0.06 (-0.04, 0.16) | **0.18 (0.08, 0.27)** |
| Ginger root | 0.2 (2.7) | 0.2 (1.3) | 0.1 (1.6) | 0.01 (-0.06, 0.08) | -0.08 (-0.16, -0.00) |
| Kale | 0.0 (0.8) | 0.1 (0.8) | 0.1 (0.7) | **0.03 (0.00, 0.06)** | **0.03 (0.00, 0.06)** |
| Leafy green | 0.3 (3.0) | 0.2 (1.6) | 0.3 (2.2) | 0.01 (-0.08, 0.09) | **0.08 (0.01, 0.16)** |
| Lettuce | 1.8 (6.9) | 1.6 (3.9) | 1.7 (4.7) | **-0.29 (-0.50, -0.09)** | -0.13 (-0.35, 0.08) |
| Mushrooms | 0.7 (4.4) | 0.6 (2.6) | 0.5 (2.9) | -0.02 (-0.14, 0.10) | -0.12 (-0.25, 0.01) |
| Non-leafy green | 0.1 (2.1) | 0.1 (1.2) | 0.1 (1.1) | 0.02 (-0.04, 0.07) | 0.00 (-0.05, 0.06) |
| Onions | 1.9 (5.8) | 2.2 (3.5) | 2.9 (5.1) | **0.41 (0.23, 0.58)** | **0.99 (0.77, 1.20)** |
| Other root vegetable | 0.3 (3.1) | 0.3 (1.4) | 0.5 (2.6) | -0.02 (-0.09, 0.06) | **0.16 (0.06, 0.26)** |
| Other vegetable | 0.5 (4.4) | 0.5 (3.0) | 0.6 (3.5) | 0.04 (-0.12, 0.19) | 0.07 (-0.08, 0.21) |
| Peppers | 2.3 (7.0) | 2.4 (4.3) | 2.8 (6.1) | 0.15 (-0.06, 0.35) | **0.53 (0.28, 0.78)** |
| Potato | 1.6 (6.4) | 1.7 (4.0) | 2.3 (5.6) | **0.20 (0.01, 0.39)** | **0.64 (0.40, 0.88)** |
| Radish | 0.2 (1.9) | 0.2 (0.8) | 0.2 (1.2) | -0.01 (-0.06, 0.04) | 0.01 (-0.05, 0.07) |
| Spinach | 0.3 (2.9) | 0.3 (1.7) | 0.3 (2.0) | -0.00 (-0.11, 0.10) | -0.05 (-0.14, 0.05) |
| Squash | 1.6 (6.0) | 2.0 (4.4) | 1.9 (4.5) | **0.58 (0.39, 0.77)** | **0.23 (0.04, 0.43)** |
| Sweet potato | 0.4 (3.5) | 0.5 (2.4) | 0.6 (3.2) | 0.07 (-0.04, 0.17) | 0.11 (-0.01, 0.24) |
| Tomato | 6.2 (12.8) | 5.1 (6.7) | 6.1 (8.8) | **-0.93 (-1.30, -0.56)** | -0.32 (-0.70, 0.07) |

CVB, cash value benefit; PLU, price look-up; USD, United States dollars; WIC, the Special Supplemental Nutrition Program for Women, Infants, and Children;

^a^ Percent of PLU-based CVB redeemed is expressed as the mean (standard deviation) percent of CVB redeemed per family (USD) in the specified category of vegetables for months during the three CVB amounts issued (T1: 9 USD/month; T2: 35 USD/month; T3: 24 USD/month) during the study period. Every observed month of redemption data was used in the calculation of means and SDs during each study period (T1: 12,032 months; T2: 4,287 months; T3: 6,121 months).

^b^ Estimate (95% confidence interval) for the percentage of the PLU-based redeemed CVB being in specified category of vegetables was determined for T2 (35 USD/month) and T3 (24 USD/month) compared to T1 (9 USD/month) in generalized estimating equations linear regression models adjusted for child race/ethnicity, sex, and age; household food insecurity and the number of household members under age 18; and calendar month (linear and quadratic). Models also accommodated clustering of monthly observations within participating children and families.

**Supplemental Table 3**. Percent of PLU-based CVB redemption that occurred in categories of fruits among WIC-participating households in Southern California before and during the CVB augmentation (June 2020-June 2022).

|  | Percent of CVB Redeemed ^a^ | | | | |
| --- | --- | --- | --- | --- | --- |
|  | T1 ^a^ | T2 ^a^ | T3 ^a^ | T2 v T1 ^b^ | T3 v T1 ^b^ |
| Apples | 3.4 (10.3) | 3.3 (6.1) | 4.7 (8.7) | 0.24 (-0.07, 0.54) | **1.29 (0.91, 1.66)** |
| Bananas | 7.1 (13.1) | 5.3 (7.4) | 5.3 (8.0) | **-1.60 (-1.97, -1.22)** | **-1.71 (-2.12, -1.30)** |
| Blackberries | 0.1 (1.6) | 0.1 (0.8) | 0.1 (1.4) | 0.02 (-0.03, 0.06) | 0.03 (-0.04, 0.09) |
| Blueberries | 0.4 (4.0) | 0.4 (2.3) | 0.3 (2.0) | -0.11 (-0.24, 0.02) | -0.10 (-0.20, 0.00) |
| Cherries | 0.6 (5.9) | 1.2 (4.8) | 0.6 (4.3) | 0.18 (-0.05, 0.41) | 0.08 (-0.07, 0.24) |
| Dragon fruit | 0.2 (3.7) | 0.3 (2.8) | 0.2 (2.5) | 0.07 (-0.05, 0.19) | 0.03 (-0.06, 0.13) |
| Grapes | 3.8 (12.4) | 3.8 (7.2) | 4.0 (9.5) | 0.30 (-0.07, 0.66) | 0.03 (-0.37, 0.44) |
| Kiwifruit | 0.5 (3.8) | 0.6 (2.8) | 0.5 (2.4) | 0.11 (-0.01, 0.23) | -0.03 (-0.13, 0.08) |
| Lemons | 0.5 (3.8) | 0.6 (3.0) | 0.7 (3.8) | **0.20 (0.06, 0.33)** | **0.18 (0.04, 0.32)** |
| Limes | 2.3 (8.4) | 2.0 (4.4) | 2.4 (6.5) | **-0.26 (-0.49, -0.03)** | -0.08 (-0.33, 0.17) |
| Mango | 1.9 (7.9) | 2.4 (5.5) | 2.2 (6.1) | **0.30 (0.03, 0.57)** | **0.50 (0.26, 0.73)** |
| Melon | 3.4 (12.5) | 4.3 (8.3) | 3.6 (9.2) | 0.29 (-0.13, 0.71) | 0.31 (-0.07, 0.70) |
| Nectarine | 0.8 (5.3) | 1.4 (4.3) | 0.8 (3.6) | **0.56 (0.37, 0.75)** | -0.02 (-0.17, 0.12) |
| Oranges | 1.8 (7.6) | 1.4 (3.7) | 1.6 (4.8) | **-0.53 (-0.76, -0.30)** | -0.17 (-0.40, 0.06) |
| Other citrus | 0.2 (2.8) | 0.2 (1.6) | 0.2 (1.9) | 0.02 (-0.06, 0.10) | -0.04 (-0.11, 0.04) |
| Other fruit | 0.4 (4.3) | 0.7 (4.1) | 0.5 (3.2) | **0.21 (0.06, 0.37)** | 0.10 (-0.02, 0.22) |
| Papaya | 0.9 (5.7) | 0.9 (3.2) | 0.8 (3.5) | -0.15 (-0.33, 0.03) | -0.07 (-0.23, 0.10) |
| Peaches | 0.6 (4.5) | 1.2 (4.1) | 0.6 (3.2) | **0.41 (0.24, 0.57)** | 0.02 (-0.10, 0.15) |
| Pears | 0.8 (4.7) | 0.7 (2.8) | 0.8 (3.2) | 0.01 (-0.11, 0.14) | -0.02 (-0.15, 0.12) |
| Pineapple | 0.7 (5.1) | 0.8 (3.0) | 0.6 (3.1) | 0.06 (-0.12, 0.23) | -0.14 (-0.30, 0.02) |
| Plums | 0.3 (2.9) | 0.4 (2.5) | 0.2 (2.0) | 0.04 (-0.06, 0.14) | -0.05 (-0.12, 0.03) |
| Raspberries | 0.3 (3.1) | 0.3 (2.1) | 0.3 (2.5) | **0.15 (0.06, 0.23)** | 0.01 (-0.08, 0.11) |
| Strawberries | 2.4 (9.9) | 2.4 (5.6) | 2.8 (7.7) | 0.14 (-0.15, 0.43) | **0.45 (0.13, 0.77)** |
| Tangerine/mandarin | 1.1 (6.2) | 0.8 (3.2) | 1.1 (4.7) | -0.03 (-0.21, 0.14) | -0.03 (-0.22, 0.17) |

CVB, cash value benefit; ; PLU, price look-up; USD, United States dollars; WIC, the Special Supplemental Nutrition Program for Women, Infants, and Children;

^a^ Percent of PLU-based CVB redeemed is expressed as the mean (standard deviation) percent of CVB redeemed per family (USD) in the specified category of fruits for months during the three CVB amounts issued (T1: 9 USD/month; T2: 35 USD/month; T3: 24 USD/month) during the study period. Every observed month of redemption data was used in the calculation of means and SDs during each study period (T1: 12,032 months; T2: 4,287 months; T3: 6,121 months).

^b^ Estimate (95% confidence interval) for the percentage of the PLU-based redeemed CVB being in specified category of fruits was determined for T2 (35 USD/month) and T3 (24 USD/month) compared to T1 (9 USD/month) in generalized estimating equations linear regression models adjusted for child race, sex, and age; household food insecurity and the number of household members under age 18; and calendar month (linear and quadratic). Models also accommodated clustering of monthly observations within participating children and families.
